# Interaural asymmetry of dynamic range: Abnormal fusion, bilateral interference, and shifts in attention

**DOI:** 10.3389/fnins.2022.1018190

**Published:** 2023-01-09

**Authors:** Sean R. Anderson, Frederick J. Gallun, Ruth Y. Litovsky

**Affiliations:** ^1^Waisman Center, University of Wisconsin-Madison, Madison, WI, United States; ^2^Department of Otolaryngology-Head and Neck Surgery, Oregon Health and Science University, Portland, OR, United States; ^3^Department of Communication Sciences and Disorders, University of Wisconsin-Madison, Madison, WI, United States; ^4^Department of Surgery, Division of Otolaryngology, University of Wisconsin-Madison, Madison, WI, United States

**Keywords:** interaural asymmetry, speech perception, binaural hearing, phonological fusion, dichotic speech, dynamic range compression, aging

## Abstract

Speech information in the better ear interferes with the poorer ear in patients with bilateral cochlear implants (BiCIs) who have large asymmetries in speech intelligibility between ears. The goal of the present study was to assess how each ear impacts, and whether one dominates, speech perception using simulated CI processing in older and younger normal-hearing (ONH and YNH) listeners. Dynamic range (DR) was manipulated symmetrically or asymmetrically across spectral bands in a vocoder. We hypothesized that if abnormal integration of speech information occurs with asymmetrical speech understanding, listeners would demonstrate an atypical preference in accuracy when reporting speech presented to the better ear and fusion of speech between the ears (i.e., an increased number of one-word responses when two words were presented). Results from three speech conditions showed that: (1) When the same word was presented to both ears, speech identification accuracy decreased if one or both ears decreased in DR, but listeners usually reported hearing one word. (2) When two words with different vowels were presented to both ears, speech identification accuracy and percentage of two-word responses decreased consistently as DR decreased in one or both ears. (3) When two rhyming words (e.g., bed and led) previously shown to phonologically fuse between ears (e.g., bled) were presented, listeners instead demonstrated interference as DR decreased. The word responded in (2) and (3) came from the right (symmetric) or better (asymmetric) ear, especially in (3) and for ONH listeners in (2). These results suggest that the ear with poorer dynamic range is downweighted by the auditory system, resulting in abnormal fusion and interference, especially for older listeners.

## 1. Introduction

Patients with bilateral severe to profound hearing loss can receive cochlear implants (CIs) to gain access to hearing. Bilateral CIs (BiCIs) improve sound source localization performance and speech understanding in noise relative to unilateral implantation (e.g., Litovsky et al., 2006). However, the extent of this benefit varies highly across patients (Litovsky et al., 2006; Mosnier et al., 2009; Yoon et al., 2011; Reeder et al., 2014; Goupell et al., 2016, 2018; Bakal et al., 2021).

Models of binaural hearing benefits based on studies completed in listeners with normal-hearing (NH) often assume that the ears act as ideal and independent channels that can be used to cancel out the masking stimulus and attend to a target of interest (e.g., Durlach, 1963). Similarly, studies presenting unrelated maskers to the ear opposite the target have suggested that listeners can ignore one ear without any decrement in performance (Cherry, 1953; Brungart and Simpson, 2002). These assumptions may not apply to patients with BiCIs, who often show marked interaural asymmetry in various aspects of auditory processing, such as speech understanding and spectro-temporal resolution. These asymmetries are likely to be produced by many different sources (Anderson, 2022). Thus, throughout this manuscript we define interaurally asymmetric hearing outcomes as any undesirable difference between the two ears to which one would answer affirmatively to the question “Does listening with your left compared to your right ear sound different?”

### 1.1. Interaural asymmetry

#### 1.1.1. Poorer ear or amount of asymmetry?

Studies of patients with BiCIs and simulations in NH suggest that interaurally asymmetric hearing outcomes may limit performance in binaural tasks (Mosnier et al., 2009; Yoon et al., 2011; Ihlefeld et al., 2015; Goupell et al., 2016, 2018; Anderson et al., 2019b,^2022^; Bakal et al., 2021). These studies assessed sensitivity to binaural cues, sound source localization, and speech understanding in background noise, and related them to asymmetry in sensitivity to temporal cues or monaural speech understanding. To address interaurally asymmetric hearing more directly, some studies first indexed or manipulated temporal fidelity in both ears, then assessed sensitivity to binaural cues. The ear with poorer temporal fidelity predicted the amount of sensitivity to binaural cues (Ihlefeld et al., 2015; Anderson et al., 2019b,^2022^). Simulations in NH used asymmetric dynamic range (i.e., amplitude modulation depth), where smaller dynamic ranges in listeners with BiCIs have resulted in poorer sensitivity to binaural cues (Ihlefeld et al., 2014; Todd et al., 2017). In these studies, performance with the *poorer ear* was predictive of the binaural benefit, suggesting that if one ear is poorly performing, it can act as a bottleneck that limits encoding of information which is used in binaural processing. Other studies evaluating speech understanding suggest that a larger relative difference *between ears* is associated with poorer benefits (Mosnier et al., 2009; Yoon et al., 2011; Goupell et al., 2016, 2018). The discrepancy in the interpretation of these findings may be due to difficultly controlling for the degree of asymmetry in studies with patients who use BiCIs, differences in the complexity of the stimuli and task, or differences between ears and limitations of the poorer ear may be at play.

#### 1.1.2. Changes in fusion or attention?

Historically, the term “fusion” has referred to many different phenomena. In the spatial hearing literature, fusion can refer to the report of a singular auditory image when a source and simulated echo are presented (Litovsky et al., 1999). In the dichotic pitch literature, fusion classically refers to the perception of a singular pitch (e.g., van den Brink et al., 1976). These subjective approaches to measuring fusion result in notoriously large amounts of variability. Moreover, spatial and pitch fusion may not always occur at the same time (Scharf, 1974). Other experimental approaches have explored fusion of speech stimuli. In the dichotic vowel literature, fusion has referred to the perception of a new vowel not corresponding to that presented in either ear (e.g., Darwin, 1981) or the reporting of only one vowel (e.g., Reiss and Molis, 2021; Eddolls et al., 2022). Similar observations can be made from the dichotic speech literature (Cutting, 1975, 1976). However, it is commonplace to report the number of items responded and interpret them in a similar way to “fusion” in these studies (e.g., Cutting, 1975; Darwin, 1981). The present experiment defines fusion as the reporting of one word, which may correspond to the left, right, both, or neither ear. We define auditory selective attention as the ability to attend to one ear (reflected in the relative weight of the left and right ear in dichotic studies). We define bilateral interference as decreased identification accuracy relative to baseline when in the presence of another stimulus in the opposite ear.

Studies have reported that, compared to a monaural condition in which both target and masker are presented to the same ear, adding a copy of the masker in the ear opposite the target speech results in improved performance for listeners with NH and BiCIs (e.g., Loizou et al., 2009; Bernstein et al., 2016; Goupell et al., 2016). It is assumed that this occurs because the masking stimuli are fused, resulting in a perceived central location within the head (i.e., spatially fused). The target speech is instead perceived on the side of the ear it is presented, resulting in unmasking. In contrast, patients with BiCIs who have marked asymmetry in speech understanding between the ears demonstrate contralateral *interference* when target speech is presented to their poorer ear (Bernstein et al., 2016; Goupell et al., 2016, 2018; Bakal et al., 2021). Listeners with a CI in one ear and NH in the other ear show the same pattern of performance (Bernstein et al., 2020). In simulations of BiCIs, contralateral interference occurs when one or both ears have poor spectro-temporal resolution (Gallun et al., 2007; Goupell et al., 2021). Two mechanisms have been proposed to drive contralateral interference in experiments where the target is presented to one ear and the masker is presented to one or both ears. The first is differences in how target and masking stimuli are perceptually segregated from one another, suggesting that they may instead be fused together (Gallun et al., 2007; Reiss and Molis, 2021). This disruptive fusion could therefore occur within the ear containing the target, across ears, or both. The second is a failure of attention, where it is more difficult to ignore the clearer stimulus (Goupell et al., 2021).

An attentional basis of contralateral interference is suggested by the finding that performance remains intact if the target is in the better ear. This has been demonstrated for listeners with BiCIs (Goupell et al., 2016, 2018; Bakal et al., 2021), listeners with one CI and one NH ear (Bernstein et al., 2016, 2020), and simulations of BiCIs in listeners with NH (Goupell et al., 2021). If contralateral interference results purely from an inability to segregate target from masker, then it should not matter whether the target is in the better or poorer ear.

Right-ear advantage has been well-documented in the auditory literature and is suspected to result from an attentional bias toward the right ear for typically developing listeners with NH (Kinsbourne, 1970; Hiscock and Kinsbourne, 2011). Another classical theory of ear advantage relates to a structural difference between the connections of the left and right ear to auditory and language processing centers (Kimura, 1967), which could be relevant for listeners with a difference in the fidelity of information represented in the left versus right ear. This may be especially relevant for listeners who experience prolonged periods of deafness, which are known to cause deterioration of the peripheral and central auditory system (e.g., Shepherd and Hardie, 2001). Interestingly, increasing age is associated with an elevated right-ear advantage (Westerhausen et al., 2015). Since most experiments concerning listeners with BiCIs tend to test older individuals, age is an important variable to account for in experiments concerning auditory spatial attention.

### 1.2. Goals and hypotheses of the present study

It is becoming clearer in the literature that processing of auditory inputs is not truly independent in each ear. Instead, information is integrated by the central auditory system and a highly efficient attentional network can be used to focus on a source of interest (e.g., Shinn-Cunningham et al., 2017). Because of the inherent connection between sound source segregation and attention, it is difficult to disentangle both processes from one another and determine how they might affect patients. While there is a right-ear advantage noted in the literature for listeners with NH, listeners with BiCIs can have considerably different speech outcomes between the ears due to many underlying factors (e.g., Litovsky et al., 2006; Mosnier et al., 2009; Goupell et al., 2018; Bakal et al., 2021). One of the major challenges of studies of interaural asymmetry in listeners with BiCIs is that large sample sizes are required to account for differences between patients. Thus, it is sometimes more practical to simulate particular sources of asymmetry in listeners with NH to determine the impact on perception. The present study simulated interaurally asymmetric dynamic range to explore the effects of degraded temporal representations on speech perception.

Our goal was to use a task that explores both fusion and auditory attention. To meet this goal, we adapted a speech perception experiment exploring a phenomenon called “phonological fusion.” In phonological fusion experiments, listeners were presented with two rhyming words to the left and right ear (Cutting, 1975, 1976). One word began with a stop consonant (e.g., /b/) and the other began with a liquid (e.g., /l/). Both words shared the same ending (e.g., /εd/), and combining the stop and liquid into a cluster would generate a word in English (e.g., bled). In the original experiments, when words were generated using natural speech productions and presented simultaneously, listeners reported hearing the fused word on approximately 30% of trials. Using synthetic speech, listeners reported hearing one word on approximately 70% of trials, which could correspond to the fused word, the word in the left or right ear, or some other word unrelated to those presented. Thus, using this paradigm, it is possible to assess whether listeners fused the percept into one word, whether listeners weighted the ears equally or unequally, and the relationship between fusion and ear-weighting on speech understanding accuracy.

In the present experiment, we assessed phonological fusion as well as closed-set speech identification of the same word or words with different vowels in each ear. This helped us evaluate a broad range of performance. It is well-known that low-frequency temporal envelope cues are essential to speech understanding in CI processing (Drullman et al., 1994; Shannon et al., 1995). The dynamic range of each electrode varies across listeners (Long et al., 2014). Smaller dynamic ranges result in poorer speech understanding (Firszt et al., 2002; Spahr et al., 2007) and binaural processing (Ihlefeld et al., 2014; Todd et al., 2017) for listeners with BiCIs. We simulated CI processing using a vocoder and manipulated the dynamic range of the speech in each ear symmetrically or asymmetrically.

The criteria used to evaluate responses (accuracy, number of words reported, response categories, and vowels) were chosen in an attempt to shed light on fusion and on the relative weight given to either ear (i.e., auditory spatial attention). In the present study, fusion was assessed primarily by the number of words being reported, consistent with recent studies (Reiss et al., 2016; Reiss and Molis, 2021; Eddolls et al., 2022), and secondarily, in Section “3.3 Phonological fusion trials,” by the proportion of phonological fusion responses consistent with classical studies (Cutting, 1975, 1976). Auditory spatial attention was assessed by whether the word(s) reported corresponded more closely to the left or right ear. Bilateral interference was assessed by the proportion of incorrect responses, most notably those that did not correspond to speech presented in either ear and could therefore not be explained by the better dynamic range of the word(s) presented. Unlike many studies concerning ear advantage, the present study asked listeners to report the word in both ears. Thus, if listeners responded with only one word, it was assumed that listeners only heard one word or they were very uncertain about what was presented to the other ear. When listeners reported two words with only one word correct, it was assumed that greater attention was being allocated toward the correctly reported ear. Finally, when one word was reported corresponding to one ear, it was assumed that the words were fused and attention was allocated to that ear. Critically, the present experiment relied on many repeated presentations to assess this and several “anchoring” conditions where both ears provided small or large dynamic range. All analyses were completed within subjects, meaning each subject acted as their own control. For a graphical description of the interpretations applied to the accuracy and number of words responded, see Supplementary Figure 1.

Three different types of trials were tested in the present experiment aiming to address different questions, and these are separated into different sections of the Results. All three kinds of trials included symmetric or asymmetric dynamic ranges. In section “3.1 Same world trials,” the same word was presented to both ears. This condition provided data concerning the characteristic errors associated with a decrease in dynamic range. Additionally, this condition allowed for the assessment of the alternative prediction: If experienced *less* fusion as dynamic range decreased in one or both ears, then listeners would report hearing one word less often. In section “3.2 Different vowel trials,” words with different vowels were presented to both ears. If listeners experienced fusion as dynamic range decreased in one or both ears and were therefore unable to attend to a single ear, then they would report hearing two words less often in these conditions and a decrease in the accuracy of correctly reporting at least one word. The latter result would occur because fusion of degraded words could result in an unintelligible word. If instead listeners were able to attend to one ear and entirely ignore the other ear, then the proportion of at least one word correct would be bounded by the symmetric dynamic range results in section “3.1 Same world trials.” In section “3.3 Phonological fusion trials,” rhyming words were presented, a subset of which could be phonologically fused to generate a new word as described in the preceding paragraph. Phonological fusion was considered to be a special case of more general fusion. If listeners experienced fusion as dynamic range decreased in one or both ears and listeners were unable to attend to only one ear, the word(s) responded would match the phonologically fused word or an incorrect word. The latter would occur because fusion of degraded words could result in a single, unintelligible word. Alternatively, if listeners were able to attend to one ear and entirely ignore the other ear, the word(s) responded would match the left, right, or both ears. Thus, sections “3.2 Different vowel trials” and “3.3 Phonological fusion trials” shed light onto the role of fusion, attention, and interference, while section “3.1 Same word trials” sheds light on the effects of the vocoder simulation.

We hypothesized that when dynamic range in both ears was decreased, listeners would experience greater fusion of words that are different from one another, decreasing the speech understanding. We further hypothesized that this would occur if the dynamic range was decreased in only one ear, listeners would also experience increased fusion and decreased speech understanding. This would be consistent with previous literature concerning discrimination of binaural cues in listeners with NH and BiCIs (Ihlefeld et al., 2015; Anderson et al., 2019b,^2022^). Alternatively, differences between the ears themselves may cause problems for listeners with BiCIs (e.g., Yoon et al., 2011). We therefore alternatively hypothesize that greater differences in dynamic range would lead to increased fusion and decreased speech understanding. We further hypothesized that listeners would exhibit right-ear advantage, weighting speech from the right ear more heavily. Thus, it was predicted that symmetrically smaller dynamic ranges would result in decreased accuracy and increased proportion of one-word responses when two words were presented. It was further predicted that listeners would correctly report more words from the right ear (i.e., right-ear advantage). It was predicted that word identification accuracy would be similar between asymmetric conditions (e.g., 100:60%) and symmetric conditions with the smaller of the asymmetric dynamic ranges (e.g., 60:60%). Alternatively, performance could reflect the difference in dynamic range between ears, where word identification accuracy in asymmetric conditions corresponds to the difference in dynamic range between the left and right ear. It was further predicted that asymmetric dynamic range conditions would bias listeners toward the better ear, where their responses would reflect the word presented to that ear (better-ear advantage).

Two groups of listeners were tested: younger NH (YNH) listeners and older NH (ONH) listeners within a similar age range to the typical CI study cohort, e.g., (Bernstein et al., 2016; Goupell et al., 2016, 2018; Baumgärtel et al., 2017; Reiss et al., 2018; Anderson et al., 2019a,^2022^; Bakal et al., 2021). Critically, aging is associated with poorer binaural and monaural temporal processing (Gallun et al., 2014; Baumgärtel et al., 2017; Anderson et al., 2019a), increased aural preference exhibited via right-ear advantage (Westerhausen et al., 2015), and decreased working memory (Roque et al., 2019). We therefore hypothesized that ONH listeners have poorer temporal processing, greater aural preference, and poorer selective attention than YNH listeners, impairing their ability to accurately identify speech and allocate attention to a better ear. It was predicted that ONH listeners would exhibit lower accuracy compared to YNH listeners across dynamic range due to poorer temporal processing. It was further predicted that ONH listeners would exhibit a higher proportion of one-word responses compared to YNH listeners due to increased aural preference. Finally, it was predicted that ONH listeners would exhibit even less accuracy in trials where two words were presented compared to YNH listeners because of increased cognitive demand.

## 2. Materials and methods

### 2.1. Listeners and equipment

**FIGURE 1:**
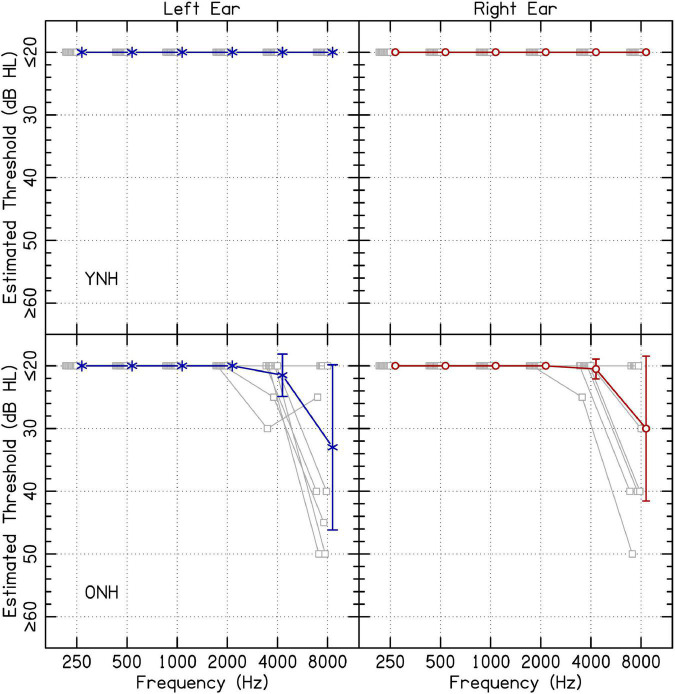
Estimated audiometric thresholds for the left and right ear. The panels on the left and right represent responses from the left and right ear, respectively. Average results are shown in blue or red, offset to the right, and error bars represent one standard deviation. Shapes offset to the left represent individual audiometric thresholds. Results from YNH and ONH listeners are shown on the top and bottom rows, respectively.

Ten YNH (20–34 years; average 26.8 years) and ten ONH listeners (52–72 years; average 59.4 years) participated in the present study. Because this experiment was completed in listeners’ homes via remote testing, experimental software was used to estimate audiometric thresholds. Listeners’ estimated audiometric thresholds are presented in Figure 1. While it is conventional to report normal hearing for YNH listeners as 20 dB HL or below, some older participants had higher estimated thresholds at 4 and 8 kHz. Audiometric responses were assessed for octave-spaced frequencies between 0.25 and 8.0 kHz using custom software in MATLAB. Sound levels in decibels hearing level (dB HL) for each frequency were determined based on the values in decibels sound pressure level (dB SPL) reported for supra-aural TDH 49/50 headphones (Frank, 1997). A conservative procedure was used to ensure that output during hearing assessment reached the desired level. The lower limit of 20 dB HL was determined based on the noise floor of the sound level meter. Sound levels were confirmed to be within 5 dB(A) of the values in dB HL for all levels except the lowest in some cases. The lowest level confirmed from the output of the sound level meter from lowest to highest frequencies were 20, 25, 30, 25, 25, and 25 dB HL, respectively. For YNH listeners, it was not possible to determine whether there were asymmetric hearing losses because the equipment could not confidently produce sound levels below 20 dB HL. For ONH listeners, participant ONH08 had a 30-dB asymmetry at 8 kHz, where the left ear had an estimated threshold of 50 dB HL and the right ear had an estimated threshold of ≤ 20 dB HL. All other ONH listeners with estimated thresholds above 20 dB HL had asymmetries ≤ 10 dB. All listeners were included in the results and analysis. All listeners spoke English as their first language. Since individual data are available with the present manuscript, analyses can be re-computed removing listeners from the dataset. All procedures were approved by the University of Wisconsin-Madison Health Sciences Institution Review Board. Listeners completed informed consent online before participation began.

Estimated audiometric thresholds were collected using custom software. The task consisted of a presentation of one, two, or three tone pips with 10-ms cosine onset- and offset-ramps. Each pip had a duration of 300 ms separated by 200-ms inter-stimulus intervals. The listener indicated the number of pips presented (three-alternative forced-choice). Testing followed standard step sizes of 10 dB-down and 5 dB-up, with a one-up, one-down adaptive rule. Levels were initiated at 70 dB HL for each frequency, beginning with 250 Hz in the left ear, increasing in frequency, and then progressing to the right ear. Threshold was estimated by a listener achieving at least two out of three presentations at the same level correct. If responses reached 20 dB HL, listeners were tested until criterion of two out of three correct. Limitations of this approach are addressed in section “4.3 Limitations.”

This experiment was conducted after the onset of the COVID-19 pandemic. Thus, testing was completed in listeners’ homes via home delivery by the experimenter. Additional applications of this approach, particularly for ONH listeners who may have mobility issues, are addressed in the discussion. Equipment consisted of noise-attenuating Sennheiser HD 280 Pro circumaural headphones, a Microsoft Surface tablet, a sound level data logger, and power supply packaged into a small box. All testing was completed using automated, custom software written in MATLAB with the Microsoft Surface in kiosk mode. Kiosk mode with limited permissions was used to ensure that the listener could not see their data or use other software on the device. Stimuli were presented at a sampling rate of 44.1 kHz. Listeners were given written setup instructions and technical assistance was available from the experimenter via remote conference on video or telephone for the duration of the experiment. Before testing began, the sound level data logger was turned on to record the sound level in the room in 1-min increments during testing, with a noise floor of 40 dB(A). All participants whose sound level data were not lost had median sound level recordings of ≤ 50 dB(A) with no more than 10 min of sound above this level during testing.

### 2.2. Stimuli

Stimuli were a subset of monosyllabic words used in previous phonological fusion experiments (Cutting, 1975, 1976). They consisted of three sets of five words. Each set had a word with a stop consonant at the onset only (bed, pay, and go), two possible liquid consonants (/l/ and /r/), and both possible stop-liquid clusters (e.g., bled and bred).

The speech corpus was produced by one male speaker from the Midwest using standard American English. During the recording process, a metronome was used to assist in generating approximately 50 tokens of each word. Two of 50 tokens per word were selected such that the corpus had roughly similar duration and pitch. The duration and pitch were then manipulated in Praat until they were approximately equal. The resulting mean and standard deviation duration was 558 ± 37 ms. The resulting mean and standard deviation pitch was 101.9 ± 1.1 Hz and all stimuli fell within one semitone. Stimuli were recorded using an M-Audio Fast Track Pro interface and AKG C5900 microphone with pop filter. Stimuli were root-mean-square (RMS) level normalized.

**FIGURE 2:**
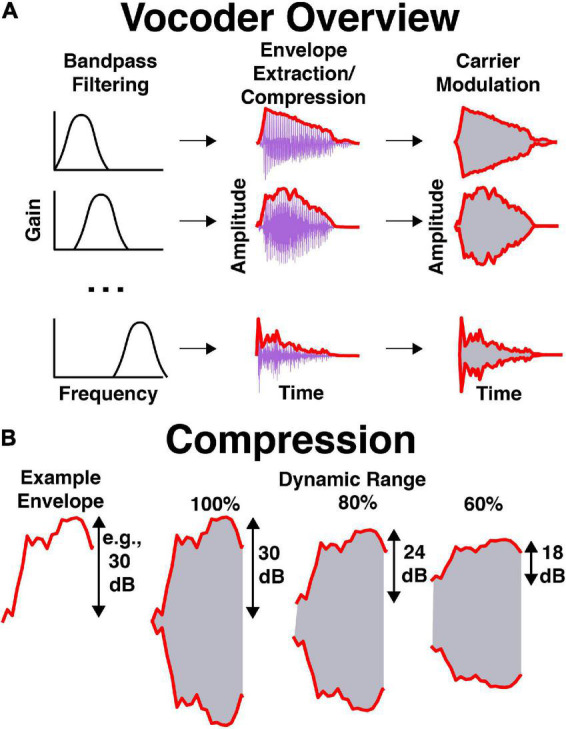
Illustration of vocoder processing. **(A)** Processing stages over time, where 16 bandpassed signals are obtained, their envelopes are extracted and compressed, noise-based carrier are modulated in amplitude, and modulated carriers are summed. **(B)** Envelope compression in dB, where the envelope is compressed to some percentage of its original range in dB. This is completed filling in the dips of the envelope, then normalizing to the original envelope level.


An illustration of stimulus processing is shown in Figure 2. Stimuli were vocoded in Praat with software that is available online.^1^ Briefly, stimuli were bandpass filtered into 16 frequency bands spaced between 250 and 8,000 Hz. Bandpass filtering was completed by multiplying stimuli in the frequency domain by Hann bands with 12 dB/octave roll-off. Frequency bands were evenly spaced and occupied equivalent cochlear space according to the Greenwood function (Greenwood, 1990). The temporal envelope was extracted using half-wave rectification and a 600-Hz low-pass filter (i.e., a Hann band from 0 to 600 Hz with 12 dB/octave roll-off). The dynamic range of the temporal envelope was manipulated by compressing the extracted envelope to some percentage of its original value in dB. For example, if the dynamic range was equal to 60% and the stimulus normally had a dynamic range of 30 dB (from a minimum level of 40 dB to maximum level of 70 dB), then the new dynamic range would be 18 dB (with a minimum level of 46 dB and a maximum of 64 dB). Therefore, the overall level remained equal between dynamic range conditions while the maximum and minimum levels within each band decreased and increased, respectively. In Praat, this was completed by performing the following procedures on the envelope: (1) adding a small positive value to shift all values above 0 in the envelope amplitude in voltage, (2) converting to dB, (3) subtracting the maximum amplitude to shift the maximum to 0 dB, (4) adding 90 to shift the maximum to 90 dB, (5) filling in the dips in the envelope proportional to one minus the dynamic range (see Eq. 1), (6) subtracting 90 and adding the original maximum to shift the maximum back to the original level in dB, (7) converting back to voltage, (8) removing the small positive value shift in voltage, (9) setting low amplitudes to 0 in voltage, (10) low-pass filtering the signal again, and (11) scaling to match the original root-mean-square amplitude of the signal. Equation 1 describes how compression was implemented in the dB domain in step 5:

(1) $$E_{c\,o\,m\,p\,r\,e\,s\,s\,e\,d}=E_{f\,u\,l\,l}+\frac{100-D\,R}{100}\,\left(M\,a\,x-E_{f\,u\,l\,l}\right)$$

where *E*_*compressed*_ and *E*_*full*_ were the time series of envelope values for the compressed and full dynmic range in dB, respectively, DR was the dynamic range in percent, and *Max* is the maximum value of the envelope in dB. In this case, *Max* was forced to be 90 dB. Thus, compression was implemented by filling in dips in the envelope, then re-scaling the amplitude to match the original level. If dynamic range was 100%, the envelope was unaltered. If dynamic range was 0%, the envelope consisted of only onsets and offsets. For additional details, see the source code available online (see text footnote 1). Low-noise noise bands (Pumplin, 1985) with bandwidths equal to the filter bandwidth were multiplied by resulting envelopes and summed across each frequency band. The resulting stimulus was RMS normalized to be equal in level to pink noise at 65 dB SPL, A-weighted [dB(A)].

### 2.3. Procedures

Listeners first confirmed that their computer set up looked like the instructions provided. Then, they confirmed to the investigator that their headphones were on the correct sides of the head. This was completed with a task measuring the side on which a 250-Hz tone of 1,000-ms duration was presented, with equal probability of being in the left or right headphone (one-interval, two-alternative forced-choice). The stimulus had 10-ms raised cosine onset- and offset-ramps. Two trials were presented at 70 dB(A). If the listener made two errors, they were instructed to reverse the headphones. If the listener made one error, they were instructed to try again. If the listener made no errors, they continued to the testing phase. Listeners completed this check again before each block of experimental trials.

In the second equipment check, listeners confirmed that they were listening via headphones and not loudspeakers using a similar task to that used by Woods et al. (2017). They were presented with three, 250-Hz tone bursts of 1,000-ms duration containing 500-ms inter-stimulus intervals. Their task was to choose the quietest burst (three-interval, two-alternative forced-choice). One tone burst was presented out of phase and at 70 dB(A). The other tones were presented in-phase at levels of 65 and 70 dB(A). Thus, if listeners were using loudspeakers and not headphones, destructive interference from the out-of-phase tone burst would reduce the sound level, making it the quietest. If instead listeners were using headphones, then the in-phase interval presented at 65 dB(A) would be the quietest. Six trials were completed. Listeners needed to achieve at least five out of six correct responses in order to progress to the next task. If they did not, they were asked to reconnect the headphones and the test was repeated. Following the second equipment check, audiograms were collected.

Next, listeners completed familiarization and a series of pre-tests. Listeners were first presented with vocoded speech and listened to any word(s) as many times as desired. A grid with the 15 stimuli in the corpus appeared on the screen. Listeners could play any word as many times as desired to the right ear. Stimuli were vocoded with 100% dynamic range. This step was completed in order to gain some familiarity with vocoded speech. Next, listeners were given a test where different tokens of the 15 words were presented one time each simultaneously to both ears and their task was to choose the word presented (i.e., 15 alternative, forced-choice). When the same word was presented to both ears, different tokens (i.e., productions) were used in the left and right ear so that listeners could not capitalize on arbitrary similarities due to using the same speech recording. If the same token had been used, then listeners may have been able to rely on similarities that do not reflect the typical variability associated with different speech productions present for all other pairs of stimuli. When different words were presented to the two ears, the token was chosen at random. Because they were also processed separately by the vocoder, the interaural correlation of the carriers in each frequency band was 0, resulting in a more diffuse sound image than if the interaural correlation were 1 (Whitmer et al., 2014). The word could not be repeated, and listeners initiated the next trial when they were ready. A minimum criterion of 10 (out of 15) correct was enforced before listeners progressed to the next task. No feedback was provided during or following testing. Next, the same test was given for stimuli vocoded with 40% dynamic range. No minimum criterion was established. Instead, the goal was simply for listeners to gain exposure to the easiest and most challenging stimuli presented during the experiment. The final pre-test consisted of pairs of stimuli with either the same word presented to each ear (10 trials) or words with different vowels presented to each ear (10 trials) using unprocessed (clean) speech. A minimum criterion of four one-word responses and four two-word responses was enforced before listeners progressed. If listeners failed to meet any criteria, they simply repeated the test until they successfully met the criteria.

Finally, listeners began experimental trials. Before each experimental block, listeners were informed that a longer block of testing was about to begin and that they could take a break if necessary. In experimental blocks, three types of trials were presented: (1) the same word using different tokens, (2) two words with different vowels, or (3) two rhyming words. Over the course of the experiment each word was tested 10 times in the “same word” trials (*n* = 150) and each possible pairing was tested in the different vowel trials (*n* = 150). When different vowels were presented, every possible combination of words was used (15 words × 10 words with different vowels = 150 combinations). The rhyming word trials consisted of two sub-types: phonological fusion and other trials. Phonological fusion trials consisted of a word beginning with a stop consonant and a word beginning with a liquid consonant, resulting in two pairs per set for the three sets, balanced so that each possible pairing was presented to the left and right ear, and each configuration repeated five times (*n* = 2 × 3 × 2 × 5 = 60). Other trials consisted of non-fusible pairs of rhyming words, with eight other pairings in each of the three sets, balanced so that each possible pairing was presented to the left and right ear, and each possible configuration repeated two times (*n* = 8 × 3 × 2 × 2 = 96). Thus, conditions with the same vowel contained a similar number of trials (*n* = 156). As an example, phonological fusion pairs for the “bed” set were: bed and led; bed and red. Thus, there were six other possible combinations: bed and bled; bed and bred; led and red; led and bled; red and bled; red and bred.

The graphical user interface included the 15 possible words, and listeners chose the word(s) they perceived during the trial. They were required to choose at least one word and were not allowed to choose more than two words. Listeners revised their decision as many times as desired and initiated the next trial by selecting “Submit” on the experiment screen. Listeners were tested with the following stimulus processing conditions: unprocessed, 100, 60, and 40% interaurally symmetric dynamic ranges, and 100:60%, 100:40%, and 60:40% interaurally asymmetric dynamic ranges. The ear with the smaller dynamic range was counterbalanced across participants. Two listeners (one YNH and one ONH) were left-handed. In asymmetric conditions, both were tested with the larger dynamic range in the left ear.

Each block had an equal number of trials from each vocoder and word-pair condition, which consisted of 315 trials for the first nine blocks and 357 trials on the final block, resulting in a total of 3,192 trials. Testing was scheduled over a 4-h period and was able to be completed by most listeners during that time, including equipment assembly and disassembly. Chance performance in the task was 1/120 as there were 120 unique response combinations (105 combinations of two words and 15 single-word responses). One listener (ONH10) had to terminate the experiment during their final block of trials, with approximately 10% of trials remaining in the block. Their data were included and weighted according to the number of trials completed.^2^ Another listener (ONH01) reported falling asleep multiple times during testing, so testing was completed over multiple days. On each trial, listeners could enter the reported words before submitting them and initiating the next trial. Thus, listener ONH01 could have entered their responses before falling asleep. Because their performance was not obviously worse than others, their data were also included.

### 2.4. Analysis

All analyses were completed using generalized (logit) linear mixed-effects analysis of variance (ANOVA) models. Version 3.5.1 of R was used with version 1.1–17 of the *lme4* package (Bates et al., 2015) to generate models and version 3.0-1 of the *lmerTest* package (Kuznetsova et al., 2017) to estimate degrees of freedom using the Kenward-Roger approximation (Kenward and Roger, 1997). Each model included a random effect associated with the listener and a fixed-effect of vocoder condition. This random effect allowed variation in mean performance due to difference between listeners to be accounted for in the model without being attributed to residual error. The ear receiving smaller dynamic range was excluded as a factor in the analysis, except in cases where ear advantage and ear bias were analyzed. The dependent variable was either: Proportion correct, proportion of one-/two-word responses, or ear advantage. Paired and post-hoc comparisons were completed using estimated marginal means with Tukey adjustments for multiple comparisons using version 1.3.0 of the *emmeans* package in R (Lenth, 2022). For the sake of brevity, z-statistics are omitted and only *p*-values are reported for paired comparisons with significant results, with non-significant pairings noted. In this case, z-tests were used because of the large sample size within each individual, where the t- and standard normal distributions become equivalent. Analyses can be replicated with the data and code provided with the present manuscript. Results were organized according to: (1) Word pairing, (2) symmetric vs. asymmetric dynamic range, (3) age (YNH vs. ONH), (4) accuracy, (5) proportion of one- or two-word responses, and (6) ear advantage (where applicable). There were 182 possible paired comparisons within each sub-section (each vocoder condition for each age group). Thus, the order of paired comparisons was determined post-hoc for aid of readability and does not necessarily reflect hypotheses or predictions, but results are interpreted in terms of predictions. Data were analyzed within the same model for each dependent variable to minimize the risk of Type I error. Analyses were re-completed excluding listeners ONH01 (who fell asleep) and listeners ONH08 (who had measurable, estimated asymmetric hearing thresholds). Any differences from the original models are reported in the results section.

## 3. Results

The goal of the present experiment was to delineate the effects of binaural speech fusion and auditory attention in simulations of BiCIs with YNH and ONH listeners. We created interaurally symmetric and asymmetric conditions with varying dynamic range. We predicted that decreasing dynamic range would result in significantly decreased accuracy, and a significant main effect or interaction showing less accuracy for ONH listeners. We further predicted that in conditions when two words were presented, the proportion of two-word responses would significantly decrease as dynamic range decreased, with a significant main effect or interaction showing fewer two-word responses for ONH listeners. Finally, we predicted that ONH listeners would show significantly more right- or worse-ear responses when dynamic ranges were interaurally symmetric or asymmetric, respectively, compared with YNH listeners. The results are separated into three sections based upon the speech presented to the listener: Same word (section “3.1 Same word trials”), words with different vowels (section “3.2 Different vowel trials”), and phonological fusion pairs (section “3.3 Phonological fusion trials”).

### 3.1. Same word trials

**FIGURE 3:**
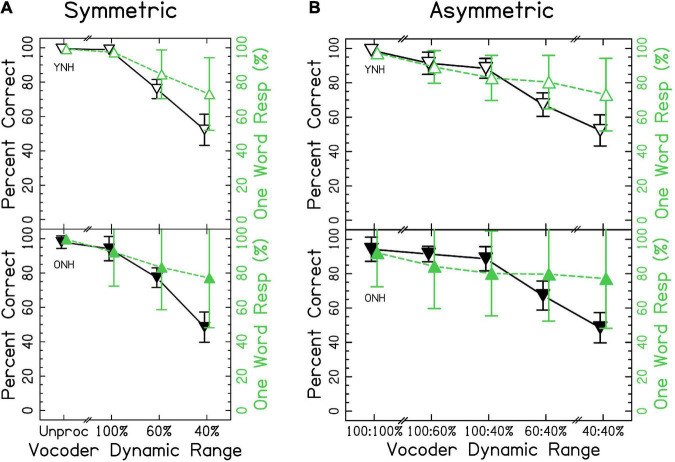
Single word accuracy and number of words responded for **(A)** interaurally symmetric, and **(B)** interaurally asymmetric vocoder conditions. The x-axis corresponds to the vocoder condition. The y-axis represents the percentage of trials with accurate, one-word responses (△ shown in black) and the percentage of one-word responses (▽ shown in green). Open and closed shapes represent YNH and ONH listeners, respectively.

Speech identification accuracy is shown in Figure 3. Accurate responses were defined as those that included only one word and when the response matched the word presented. When the data were fit with the mixed-effects ANOVA, model diagnostics revealed substantial deviation from the assumption that residuals were normally distributed due to one outlying observation (listener ONH03 in the unprocessed condition). This observation was removed from analysis, which resolved the issue though this resulted in no change in conclusions. The results of the ANOVA demonstrated a significant effect of vocoder condition [χ^2^(6) = 2705.502, *p* < 0.0001], with smaller dynamic ranges resulting in less accuracy, consistent with the hypotheses. Age group was not significant [χ^2^(1) = 0.268, *p* = 0.604]. However, there was a significant vocoder condition × age group interaction [χ^2^(6) = 46.421, *p* < 0.0001] that is investigated further in the sections that follow. Confusion matrices describing errors in interaurally symmetric conditions are shown in Supplementary Figure 2. Two patterns are obvious: (1) Few vowel errors occurred, and (2) for 60 and 40% dynamic range, the most common confusion was reporting a stop-liquid cluster when a liquid was presented. For more details, see section “4.2 Ear advantage” of the discussion.

We wanted to rule out the possibility that, as dynamic range was decreased, listeners began to perceive multiple words. The proportion of one-word responses is also shown in Figure 3. Results of the ANOVA demonstrated a significant effect of vocoder condition [χ^2^(6) = 781.616, *p* < 0.0001] but not age group [χ^2^(1) = 0.042, *p* = 0.838] on percent correct. There was a significant vocoder condition × age group interaction [χ^2^(6) = 31.669, *p* < 0.0001].

#### 3.1.1. Interaurally symmetric conditions

Figure 3A shows results from the interaurally symmetric conditions. Consistent with the hypotheses, the percentage of words correctly identified was significantly higher for the larger dynamic range in all pairs of symmetric vocoder conditions for both groups [*p* < 0.05–0.0001]. It was of interest to determine whether the vocoder condition × age group interaction was driven by differences between age groups at selected dynamic ranges. Pairwise comparisons with symmetric vocoder conditions showed no significant differences between YNH and ONH listeners in matched vocoder conditions, suggesting that the interaction was driven by the asymmetric conditions or differences in effects within groups.

Similarly, for proportion of one-word responses, post-hoc comparisons showed no significant differences between age groups in matched vocoder conditions. There were significant differences in proportion of one-word responses between all pairs of symmetric vocoder conditions for both groups [*p* < 0.01–0.0001]. In all cases, the proportion of one-word responses was higher for the larger dynamic range.

#### 3.1.2. Interaurally asymmetric conditions

When analyzing the interaurally asymmetric conditions, it was of interest to determine whether accuracy reflected maximum dynamic range (i.e., better ear), the mean dynamic range, the minimum dynamic range (i.e., worse ear), or the difference in dynamic ranges (i.e., degree of asymmetry). We predicted that accuracy would reflect the poorer ear or degree of asymmetry, with poorer performance for ONH listeners on average. For example, with 100:60% dynamic range, the maximum was 100%, the mean was 80%, the minimum was 60%, and the difference was 40%. Figure 3B shows accuracy for the interaurally asymmetric conditions, bounded by interaurally symmetric conditions with the largest and smallest dynamic ranges. Contrary to primary and alternative hypotheses, results support the notion that accuracy reflected the mean dynamic range between ears. Paired comparisons revealed differences in the level of significance between YNH and ONH listeners. In the YNH group, speech identification accuracy was significantly higher for the larger mean dynamic range [*p* < 0.05–0.0001]. In the ONH group, there was no significant difference between the 100:40% compared to 60:60% [*p* = 0.446] and the 100:60% compared to the 100:40% [*p* = 0.128] conditions, but all others were significant. In other words, while the overall patterns were the same, differences between asymmetric conditions tended to be less pronounced for ONH listeners.

#### 3.1.3. Summary

These results suggest that speech identification, and to a lesser extent fusion, of the same word reflect the mean dynamic range across-ears (e.g., mean of 100 and 40% is 70%), in disagreement with our predictions that the worse ear or degree of asymmetry would predict accuracy. In further disagreement with our hypotheses, there was no consistent effect of age group when comparing at the same dynamic ranges between groups. By definition, if a listener responded with two words, their response was scored as incorrect. Thus, the highest level of accuracy was defined by the proportion of one-word responses. Based on the analysis, Figure 3, and Supplementary Figure 2, the results suggest that decreases in accuracy did not strictly reflect reporting more words, rather that listeners made systematic errors.

### 3.2. Different vowel trials

**FIGURE 4:**
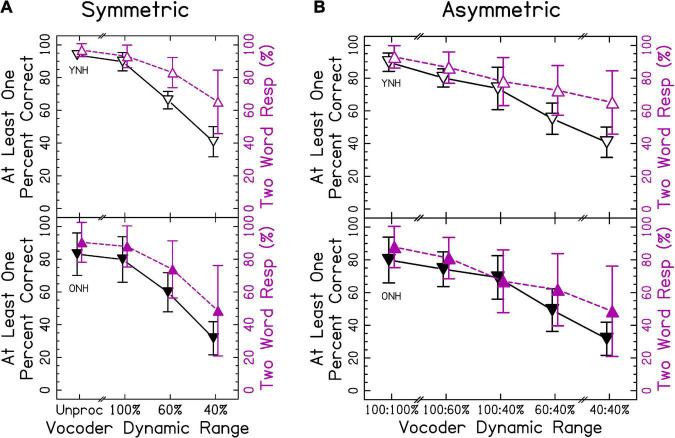
Different vowel accuracy and number of words responded for **(A)** interaurally symmetric, and **(B)** interaurally asymmetric vocoding conditions. The x-axis corresponds to the vocoder condition. The y-axis represents the percentage of trials with at least one word accurately identified (△ shown in black) and the percentage of two-word responses (▽ shown in purple). Open and closed shapes represent YNH and ONH listeners, respectively.

In sections “3.2 Different vowel trials” and “3.3 Phonological fusion trials,” different words were presented to each ear. Speech identification accuracy is shown in Figure 4. In this case, the accuracy represents the probability of correctly reporting at least one word. We predicted that accuracy would decrease as the dynamic range decreased for both groups. Presenting two words simultaneously was more cognitively demanding. Accordingly, we predicted that ONH listeners would show poorer accuracy than YNH listeners. This increased difficulty was more likely to elicit ear advantage, where attention to one ear was prioritized. Thus, our definition of accuracy was formulated to determine whether information in at least one ear was preserved. Additionally, if listeners were able to listen to each ear independently, then the accuracy (at least one correct) in section “3.2 Different vowel trials” should be equal to the accuracy in section “3.1 Same word trials.” The ANOVA revealed significant effects of vocoder condition [χ^2^(6) = 2477.006, *p* < 0.0001] where smaller dynamic ranges resulted in less accuracy, consistent with our hypotheses. There was also a significant effect of age group [χ^2^(1) = 5.104, *p* < 0.05], with ONH listeners having a lower percentage of correct responses consistent with our hypotheses. There was also a significant vocoder condition × age group interaction [χ^2^(6) = 55.975, *p* < 0.0001] that is explored further in the next sections. Vowel confusion matrices describing errors in interaurally symmetric conditions are shown in Supplementary Figure 3. It is important to note that the effect of age changed from *p* = 0.040–0.085 if listeners ONH01 (who fell asleep) and listener ONH08 (who had asymmetric, estimated hearing thresholds) were excluded from analysis. No other changes in statistical inference occurred.

We wanted to titrate the types of errors made in each symmetric vocoder condition. There were a total of 75 two-word response combinations as well as 15 one-word response possibilities. Thus, a confusion matrix would be difficult to show with every possible combination. Instead, capitalizing on the small number of vowel errors made with single word trials, Supplementary Figure 3 shows vowel confusion matrices for interaurally symmetric vocoder trials. There were only three possible vowel combinations on each trial, but single vowel responses were also considered. As can be seen from this figure, vowel errors were very rare. When listeners reported a single vowel, this usually corresponded to one of the vowels presented in one ear. The /ε/ and /eI/ pairs were the most likely to result in singular vowel responses. This may have to do with the fact that the /ε/ set had an additional /d/ cue at the end of each word.

Compared with single word trials, there was a much higher correspondence between the number of words reported and the accuracy in reporting at least one word correct, consistent with our hypotheses that decreased dynamic range would result in fusion. When percentage of two-word responses was the dependent variable in the ANOVA, there was a significant effect of vocoder condition [χ^2^(6) = 1658.486, *p* < 0.0001], with smaller dynamic ranges resulting in greater one-word responses consistent with our hypotheses. There was no effect of age group [χ^2^(1) = 1.194, *p* = 0.274], inconsistent with our hypotheses. There was a significant vocoder condition × age group interaction [χ^2^(6) = 21.421, *p* < 0.01], which is explored further in the sections “3.2.1 Interaurally symmetric conditions” and “3.2.2 Interaurally asymmetric conditions.”

#### 3.2.1. Interaurally symmetric conditions

Figure 4A shows results from the interaurally symmetric conditions. Consistent with the hypotheses, the percentage of trials with at least one word correctly identified was significantly higher for the greater dynamic range between all pairs of symmetric vocoder conditions for both groups [*p* < 0.01–0.0001], except for the unprocessed and 100% dynamic range conditions for ONH listeners [*p* = 0.540]. Further consistent with our hypotheses, pairwise comparisons with symmetric vocoder conditions showed that speech identification accuracy was significantly greater for YNH compared to ONH listeners in the unprocessed [*p* < 0.0001] and 100% [*p* < 0.01] conditions, but not the 60% [*p* = 0.975] and 40% [*p* = 0.584] conditions.

Similar to identification accuracy and consistent with our hypotheses, the proportion of two-word responses was significantly higher for the larger dynamic range in all pairs of symmetric vocoder conditions for both groups [*p* < 0.001–0.0001], except for the unprocessed and 100% dynamic range conditions for ONH listeners [*p* = 0.563]. For proportion of two-word responses and inconsistent with our hypotheses, pairwise comparisons showed no significant differences between groups in matched vocoder conditions.

#### 3.2.2. Interaurally asymmetric conditions

Figure 4B shows accuracy for the interaurally asymmetric conditions, bounded by interaurally symmetric conditions with the largest and smallest dynamic ranges. Results support that accuracy was reflected by the mean dynamic range between ears, inconsistent with our hypotheses that the poorer ear or degree of asymmetry would predict performance. Pairwise comparisons revealed differences in the level of significance between YNH and ONH listeners. In both groups, most speech identification accuracy was significantly greater for the higher mean dynamic range [*p* < 0.05–0.0001]. In the ONH group, there was no significant difference between the 100:40% compared to 60:60% [*p* = 0.109] conditions.

The proportion of two-word responses was similar to that observed for speech identification accuracy, with some slight differences between the YNH and ONH groups. In both groups, the proportion of two-word responses was significantly greater for the higher mean dynamic range [*p* < 0.05–0.0001], except for the 100:60% compared to 60:60% [*p* = 0.288] in YNH listeners and 100:40% compared to 60:40% [*p* = 0.077] conditions in ONH listeners. There was one interesting exception to this pattern. For both the YNH and ONH groups, the proportion of two-word responses was significantly greater for the 60:60% compared to 100:40% [*p* < 0.0001] conditions. This suggests that, listeners reported one word in the cases with the largest amount of asymmetry (100:40%) compared to when stimuli were symmetric and poorly represented (60:60%), inconsistent with our hypotheses.

#### 3.2.3. Ear advantage

Figure 4 suggests that there was a strong correspondence between speech identification accuracy and proportion of two-word responses, despite accuracy being based upon correctly reporting the word presented to either ear. Additionally, Supplementary Figures 2, 3 demonstrate that listeners were unlikely to make a vowel error even with small dynamic ranges. Thus, the vowel reported likely corresponds to the ear to which the listener was allocating attention. The left- and right-ear advantage was explored by evaluating the proportion of vowels reported from the left or right ear when only one word was reported. We hypothesized that listeners would show a right-ear advantage in symmetric conditions that increased with decreasing dynamic range, a better-ear advantage in asymmetric conditions, and that right- or worse-ear advantage would be greater in ONH compared to YNH listeners.

**FIGURE 5:**
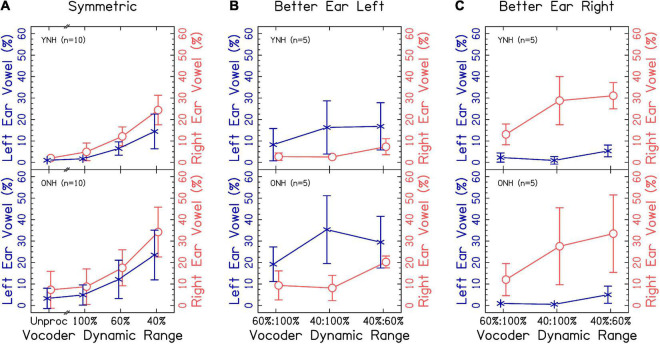
Vowel responses by ear for different vowel trials for **(A)** interaurally symmetric or **(B,C)** interaurally asymmetric vocoder conditions. The x-axis corresponds to the vocoder condition. The y-axis corresponds to the percentage of trials where the vowel of the response was one word and came from the left (× shown in blue) or right (○ shown in orange).

Results from Figure 5A suggest a modest right-ear advantage across interaurally symmetric vocoder conditions, with the smallest dynamic ranges resulting in the greatest ear advantages. A mixed-effects ANOVA revealed significant fixed-effects of ear [χ^2^(1) = 196.565, *p* < 0.0001], with a greater proportion of right ear responses consistent with our hypotheses. There was also a significant effect of vocoder condition [χ^2^(3) = 1329.536, *p* < 0.0001], with smaller dynamic ranges resulting in increased ear advantage responses consistent with our hypotheses. There was also a significant effect of age group [χ^2^(1) = 6.018, *p* < 0.05], with ONH listeners showing greater ear advantage consistent with our hypotheses. There was also a significant vocoder condition × age group interaction [χ^2^(6) = 19.436, *p* < 0.001]. Pairwise comparisons showed that ONH listeners had significantly more responses compared to YNH listeners in only the unprocessed condition [*p* < 0.0001]. There was no significant ear × vocoder condition [χ^2^(3) = 4.938, *p* = 0.176], ear × age group [χ^2^(1) = 3.825, *p* = 0.050], or three-way [χ^2^(3) = 2.528, *p* = 0.470] interactions. The model with dependent variable proportion correct did not converge if listeners ONH01 and ONH08 were excluded from analysis.

Figures 5B, C show the same results for interaurally asymmetric vocoder conditions, with the better (i.e., ear with larger dynamic range) or worse ear. In order to analyze the data, the factor “ear” was re-coded from left or right to better or worse. A mixed-effects ANOVA revealed significant fixed-effects of ear [χ^2^(1) = 856.650, *p* < 0.0001], with a higher proportion of better-ear responses consistent with the hypotheses. There was also a significant effect of vocoder condition [χ^2^(2) = 287.988, *p* < 0.0001], with smaller dynamic ranges resulting in larger ear advantage consistent with our hypotheses. There was not a significant effect of age group [χ^2^(1) = 3.419, *p* = 0.064], inconsistent with our hypotheses. There were significant ear × vocoder condition [χ^2^(2) = 82.685, *p* < 0.0001] and ear × age group [χ^2^(1) = 11.016, *p* < 0.001] interactions, which will be addressed in the next paragraph. Vocoder condition × age group [χ^2^(2) = 0.730, *p* = 0.694] and three-way [χ^2^(2) = 0.196, *p* = 0.906] interactions were not significant.

Pairwise comparisons revealed that there were significantly more better-ear responses in the 100:40% compared to 100:60% [*p* < 0.0001] and 60:40% compared to 100:60% [*p* < 0.0001], but not the 60:40% compared to 100:40% [*p* = 0.983] conditions. In contrast, there were significantly more worse-ear responses in the 60:40% compared to 100:60% [*p* < 0.0001] and 60:40% compared to 100:40% [*p* < 0.0001] conditions, but not between the 100:40% and 100:60% [*p* = 0.507] conditions, which we did not predict. The ONH group had significantly more worse-ear responses than the YNH group [*p* < 0.05], but no difference between the better-ear responses [*p* = 0.396], consistent with our hypotheses. There were significantly more better-ear compared to worse ear responses [*p* < 0.0001] in all three vocoder conditions.

#### 3.2.4. Summary

The degree of accuracy and proportion of two-word responses decreased in a similar fashion as dynamic range decreased, consistent with our hypotheses. Accuracy was lower for ONH compared to YNH listeners in the unprocessed and 100:100% dynamic range condition, but the proportion of two-word responses was not different between groups. These results suggest that speech identification and fusion of the words with different vowels reflect the average dynamic range across the ears. They further suggest that the accuracy of speech identification might be mediated by fusion in the larger dynamic range conditions. When dynamic range was small in one ear (60 and/or 40%), the correspondence between accuracy and proportion of two-word responses was less strong but still present. This implies that listeners were more likely to report hearing one word, and more likely to have that word be inaccurate, when speech was degraded in one or both ears. This is intuitive, since an effective strategy may be to ignore the poorer ear. The results demonstrate that poor dynamic range in general impairs access to speech in both ears (evidenced by significantly poorer speech understanding for 100:60% and 100:40% compared to 100:100%). Thus, it was of particular interest to explore the probability of responding with a correct response in the left or right ear. In contrast to our hypotheses, there were only significantly more right-ear responses for ONH listeners in the unprocessed condition. Notably, there were significantly more *worse* ear responses for ONH listeners when dynamic range was asymmetric. This result may reflect an inability to ignore the right ear for ONH listeners, even when it has a smaller dynamic range. Additionally, the results suggest that interaurally asymmetric dynamic range interacts with right-ear advantage, with larger right-ear advantage when the right ear has greater dynamic range (Figures 5B, C though this was not tested statistically).

### 3.3. Phonological fusion trials

**FIGURE 6:**
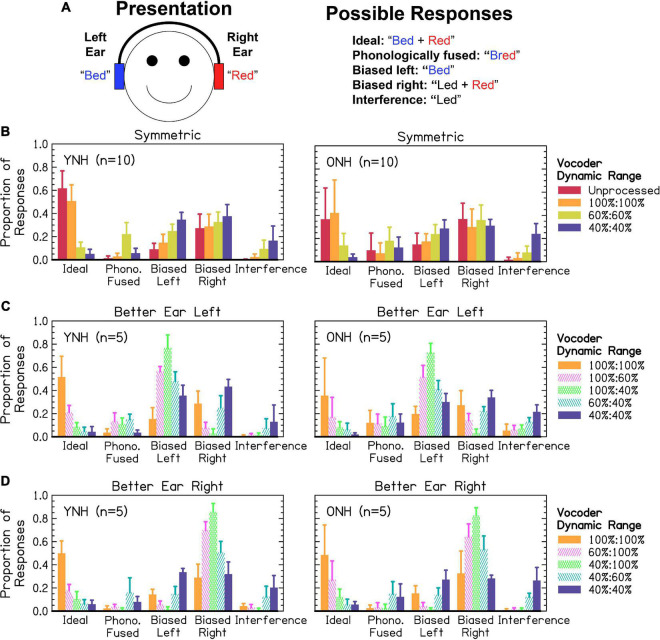
**(A)** Response categories for one example phonological fusion trial. **(B–D)** Relative frequency of response categories observed by listeners in **(B)** symmetric and **(C,D)** asymmetric dynamic ranges. The x-axis corresponds to the response category from **(A)**. The y-axis corresponds to the proportion of responses. The color and pattern represent the vocoder condition given in the figure legend and arranged from highest to lowest mean dynamic range. The height of bars represents the mean across listeners. Error bars represent standard deviation of the mean across listeners.

Figure 6A shows an example trial from a phonological fusion trial and how responses were scored. Ideal responses required listeners to respond with two words, with both words correct. Phonologically fused responses required listeners to respond with one word containing the stop-liquid cluster of the stop and liquid presented. Biased left required listeners to respond with one or two words, with only the word from the left ear correct. The same was true for biased right except that the word reported was presented to the right ear. Interference responses required listeners to respond with one or two words corresponding neither to the word presented in the left, right, or phonologically fused word. These possibilities can be viewed as a continuum from best-case (independent channels to which attention can be allocated or ideally linked channels where information is shared) to worse-case scenario (interfering channels to which attention cannot be effectively allocated). Figures 6B–D shows responses from participants. We predicted that the proportion of ideal responses would be highest for the largest dynamic ranges, lowest for the smallest dynamic ranges, and the worse ear would predict ideal and interference responses in asymmetric conditions. We further predicted that the proportion of interference responses would increase as dynamic range decreased. We predicted that asymmetric dynamic ranges would result in increased responses biased toward the better ear. Finally, we predicted that ONH listeners would exhibit fewer ideal, more interference, and more biased responses compared to YNH listeners. Listeners responded with one word on 0–100% of trials, with a mean of 58% and standard deviation of 26%. While the original studies on phonological fusion (Cutting, 1975, 1976) showed phonological fusion responses (i.e., one word with a stop and liquid cluster) in approximately 30% of trials, the relative frequency in the present study was lower. This will be explored further in the discussion. Data were analyzed in two separate sections addressing: (1) the proportion of ideal and interference responses, representing the best- and worst-case scenarios for listeners, and (2) the relative bias toward responding correctly from the left and right (symmetric) or better and worse (asymmetric) ears.

#### 3.3.1. Ideal and interference responses

When data were fit with the mixed-effects ANOVAs, residuals were curvilinear and not normally distributed. Diagnostic plots suggested that the residuals may have been Cauchy distributed for the proportion of ideal responses. When a cauchit (rather than logit) ANOVA was used based on the pattern of residuals post-hoc, the model estimating ideal responses improved considerably, resulting in normally distributed residuals and the removal of curvilinearity. Including a random effect of vocoder condition led to normally distributed residuals for the proportion of interference responses. There was still some slight overestimation at the smallest proportions of ideal and interference responses. In both cases, this could have led to decreased power.

The results of the cauchit ANOVA demonstrated significant effects of vocoder condition [χ^2^(6) = 458.372, *p* < 0.0001], with smaller dynamic ranges resulting in fewer ideal responses consistent with the hypotheses. There was no significant effect of age group [χ^2^(1) = 2.490, *p* = 0.115], inconsistent with our hypotheses. There was a significant vocoder condition × group interaction [χ^2^(6) = 43.4445, *p* < 0.0001], which is addressed in the next sections. A logit ANOVA including random effect of vocoder condition showed significant effects of vocoder condition [χ^2^(6) = 81.024, *p* < 0.0001], with smaller dynamic ranges resulting in more interference responses consistent with the hypotheses. There was also a significant effect of age group [χ^2^(1) = 5.717, *p* < 0.05], with ONH listeners demonstrating more interference responses consistent with the hypotheses. There was no significant vocoder condition × group interaction [χ^2^(6) = 9.784, *p* = 0.134]. The model with dependent variable interference did not converge if listeners ONH01 and ONH08 were excluded from analysis.

Interaurally symmetric conditions are shown in Figure 6B. The proportion of ideal responses was not significantly different between YNH or ONH in any of the symmetric vocoder conditions, inconsistent with the hypotheses. For YNH listeners, there were significant differences between all vocoder conditions [*p* < 0.01–0.0001] except 60 and 40% dynamic range [*p* = 0.211]. For ONH listeners, there were significant differences between all vocoder conditions [*p* < 0.05–0.0001], except unprocessed and 100% dynamic range [*p* = 0.527]. The proportion of interference responses significantly increased as dynamic range decreased in both age groups [*p* < 0.01–0.0001], consistent with the hypotheses. There was no difference between the unprocessed and 100% dynamic range condition [*p* = 0.346]. The proportion of interference responses was significantly higher for ONH compared to YNH listeners, consistent with the hypotheses.

Figures 6C, D show the proportion of ideal and interference responses in asymmetric conditions, bounded by interaurally symmetric conditions with the largest and smallest dynamic ranges. In general, the worse ear was predictive of the proportion of ideal responses, consistent with the hypotheses. For YNH listeners, proportion of ideal responses was lowest when at least one ear had 40% dynamic range. There were no significant differences between all conditions where one ear had 40% dynamic range. There was also no significant difference between the 100:40 and 60:60% dynamic range conditions [*p* = 1.000]. For greater dynamic ranges (60–100%), the proportion of ideal responses was highest for conditions with the greatest mean dynamic range [*p* < 0.05–0.0001]. For ONH listeners, there no difference between all vocoder conditions with equivalent minimum dynamic ranges, consistent with the hypotheses. There were two exceptions to this, (1) the 100:60% dynamic range led to significantly more ideal responses than the 60:60% [*p* < 0.01] condition, and (2) there was no difference between the 60:60% and 100:40% [*p* = 0.464] conditions. The better ear was predictive of the proportion of interference responses, inconsistent with the hypotheses. Accordingly, there were no significant differences between 100:100% and 100:60% [*p* = 0.987], 100:100% and 100:40% [*p* = 1.000], 100:60% and 100:40% [*p* = 0.994], or 60:60% and 60:40% [*p* = 0.747]. All other pairs (excluding unprocessed) were significantly different [*p* < 0.01–0.0001].

#### 3.3.2. Ear bias

Results from Figure 6B show a right ear bias across interaurally symmetric vocoder conditions, with the largest dynamic ranges resulting in the greatest ear bias, inconsistent with the hypotheses. A mixed-effects ANOVA revealed significant fixed-effects of ear [χ^2^(1) = 150.792, *p* < 0.0001], with the right ear resulting in a greater proportion of responses consistent with the hypotheses. There was also a significant effect of vocoder condition [χ^2^(3) = 96.242, *p* < 0.0001]. The left ear bias increased as dynamic range decreased [*p* < 0.05–0.0001], where the right ear bias was not significantly different between any pairs of conditions inconsistent with the hypotheses. This was reflected by a significant ear × vocoder condition interaction [χ^2^(3) = 69.913, *p* < 0.0001]. There was not a significant effect of age group [χ^2^(1) = 0.247, *p* = 0.619]. However, there was a significant vocoder condition × age group interaction [χ^2^(3) = 31.953, *p* < 0.0001]. Older NH compared to YNH listeners had significantly greater bias responses in the unprocessed [*p* < 0.05] but no other conditions, inconsistent the hypotheses. Ear × age group [χ^2^(1) = 0.002, *p* = 0.962] and three-way [χ^2^(3) = 1.904, *p* = 0.593] interactions were not significant. For YNH listeners, smaller dynamic ranges resulted in significantly greater bias responses [*p* < 0.01–0.0001] except for the unprocessed compared to 100% [*p* = 0.098] condition. For ONH listeners, bias was only significantly greater in the 40% compared to 100% [*p* < 0.01] and 60% compared 100% [*p* < 0.01] conditions.

Results from Figures 6C, D show a strong bias toward the better ear, especially in the cases of largest asymmetry. The mixed-effects ANOVA revealed a significant fixed-effect of ear [χ^2^(1) = 1070.238, *p* < 0.0001], with a higher proportion of better ear bias consistent with the hypotheses. There was also a significant effect of vocoder condition [χ^2^(2) = 91.005, *p* < 0.0001], with greater degree of asymmetry predicting the portion of better ear bias, inconsistent with the hypotheses. There was not a significant effect of age group [χ^2^(1) = 1.936, *p* = 0.164], inconsistent with the hypotheses. There was a significant ear × vocoder condition interaction [χ^2^(2) = 302.531, *p* < 0.0001], reflected in the increased bias toward the better ear with increasing asymmetry inconsistent with our hypotheses. There were not significant ear × age group [χ^2^(1) = 2.086, *p* = 0.149] or vocoder condition × age group [χ^2^(2) = 3.821, *p* = 0.148] interactions, further suggesting no differences between age groups and inconsistent with the hypotheses. Paired comparisons revealed that all pairs of ear and vocoder conditions were significantly different [*p* < 0.0001]. Most better-ear responses occurred for the 100:40%, followed by the 100:60%, followed by the 60:40% conditions. The greatest amount of worse-ear responses occurred for the 60:40%, followed by the 100:60%, followed by the 100:40% conditions.

#### 3.3.3. Summary

Consistent with our hypotheses, when dynamic range was symmetric, listeners tended to report at least one word incorrectly as dynamic range decreased (i.e., fewer “ideal” responses), shifting their responses to the word presented in the left ear or a word that was not presented (i.e., an “interference” response). Responses from the word in the right ear remained consistent across conditions. When dynamic range was asymmetric, opposite and opposing effects were observed for ideal versus interference and better-ear versus worse-ear responses. The proportion of ideal responses decreased as the dynamic range in the worse ear decreased. The proportion of interference responses decreased as the dynamic range in the better ear increased. Better-ear responses increased and worse-ear responses decreased as the degree of interaural asymmetry increased, consistent with the notion that listeners attended to the better ear when dynamic range was asymmetric. We further hypothesized that ONH listeners would exhibit more interference responses, which was confirmed by the results, and more ear bias, which was refuted by the results. Like different vowel trials, ONH listeners only exhibited greater bias toward the right ear compared to YNH listeners in the unprocessed condition, which was inconsistent with our hypotheses.

## 4. Discussion

Patients with BiCIs often experience substantial differences in hearing outcomes between their ears. While bilateral implantation generally improves speech understanding in noise relative to unilateral implantation, there are some conditions under which bilateral hearing is not beneficial, and listeners experience contralateral interference from the better ear (Bernstein et al., 2016, 2020; Goupell et al., 2016, 2018). Thus, addressing the mechanisms leading to contralateral interference may play a key role in maximizing bilateral outcomes. Two putative mechanisms have been proposed in the literature related to the basis of contralateral interference. The first supposes that it results from a failure of sound source segregation cues to form distinct auditory objects (Gallun et al., 2007; Reiss et al., 2016; Reiss and Molis, 2021). The second supposes that contralateral interference results from a failure to allocate attention away from the source with greatest spectro-temporal fidelity and toward a more degraded sound source (Goupell et al., 2016, 2021).

Results from the present experiment suggest that poor segregation of sound sources and compromised auditory spatial attention both play a role and may interact with one another when temporal information is symmetrically or asymmetrically degraded. Listeners showed an increased number of one-word responses when words with dichotic vowels were presented as stimuli became increasingly symmetrically or asymmetrically degraded. As stimuli became increasingly degraded in one or both ears, listeners also tended to report inaccurate word(s). Inaccuracies occurred despite evidence that listeners shifted attention toward the right or left ear when stimuli were symmetrically degraded and toward the better ear when stimuli were asymmetrically degraded. In particular, the present experiment challenges the assumption of classical theories in binaural hearing that the ears act as independent channels. If both ears were independent, then listeners should have attended to the better ear in asymmetric dynamic conditions and correctly reported that word. This would have resulted in similar accuracy in sections “3.1 Same word trials” and “3.2 Different vowel trials,” and no interference responses in section “3.3 Phonological fusion trials.” Instead, listeners showed substantially poorer accuracy in section “3.2 Different vowel trials” compared to section “3.1 Same word trials” and monotonically increasing interference as dynamic range decreased in section “3.3 Phonological fusion trials.” Moreover, listeners showed marked right-ear advantage when stimuli were symmetrically degraded.

### 4.1. Object-based auditory attention

Auditory attention is thought to be a process with serial and parallel stages (Bregman, 1994; Shinn-Cunningham, 2008). First, brief and similar spectral components are grouped into auditory streams. These streams compete for attention over longer periods of time, and top-down attention is allocated to a source of interest via source segregation cues. Finally, information (e.g., language content) can be extracted from sources of interest. This is a simpler process for stimuli that occur in quiet with an unobscured onset and offset time. However, real-world listening often occurs in complex auditory environments with competing background noise and ambiguous onset and offset times. It is suspected that listeners maintain an internal perceptual model that can be updated according to new sensory information to efficiently and robustly complete this process (e.g., Rao and Ballard, 1999). Increasingly complex stimulus features are suspected to be extracted at later stages of sensory processing. These features are the product of interactions between internal predictions and incoming sensory input. Thus, for listeners who receive compromised or ambiguous sound source segregation cues, it is likely that the ability to maintain internal predictions, represent sound features, and allocate attention is compromised.

Listeners with BiCIs show patterns of behavior indicating that they might fuse unrelated auditory information. For example, listeners perceive a singular pitch percept over a large disparity of electrodes between ears, corresponding to frequency differences up to one octave (Reiss et al., 2018). Abnormally large fusion ranges for interaural place-of-stimulation differences have been proposed by Reiss et al. (2015) as an adaptive process associated with the large degrees of mismatch due to differences in degree of insertion for listeners with BiCIs (Goupell et al., 2022). Simulations in NH suggest that interaural mismatches in place-of-stimulation result in poorer spatial fusion (Goupell et al., 2013) and speech fusion (Aronoff et al., 2015; Staisloff et al., 2016). Accordingly, listeners with BiCIs perceive a singular spatial image (as opposed to multiple perceived locations) over large interaural electrode disparities, even as the impact of binaural cues on perceived intracranial perception decreases (van Hoesel and Clark, 1995, 1997; Long et al., 2003; Kan et al., 2013, 2019). Listeners with BiCIs will fuse stimuli presented with interaural timing differences up to several milliseconds (van Hoesel and Clark, 1995, 1997) and very large interaural timing differences (∼2 ms) are needed to achieve maximum intracranial lateralization (Litovsky et al., 2010; Baumgärtel et al., 2017; Anderson et al., 2019a).

It has been suggested that listeners with hearing loss struggle in noise because of an inability to accurately segregate speech sounds, leading them to fuse unrelated words (e.g., Reiss et al., 2016; Reiss and Molis, 2021; Eddolls et al., 2022). Results from the present study support these findings. In particular, there was a high correspondence between the proportion of two-word responses and accuracy reporting at least one word correctly when vowels differed (Figure 4) as well as a higher proportion of interference (Figure 6) when stimuli were temporally degraded. The present study used less conservative criteria for accuracy compared to other fusion studies using synthesized vowels (Reiss and Molis, 2021; Eddolls et al., 2022). While the present study always presented speech bilaterally, we can draw some conclusions from the symmetric versus asymmetric conditions. In particular, results from listeners with hearing loss have shown that stimulating each ear individually results in a different pattern of performance compared to bilateral stimulation for some listeners, presumably because of underlying differences between ears or poor overall representations of spectro-temporal cues (Reiss et al., 2016). The present study showed that small, symmetric or asymmetric dynamic range resulted in a decrease of accuracy in reporting at least one word correct when two were presented compared to symmetric conditions with only one word. This suggests that dichotic speech with poor dynamic range leads to different perception than diotic or monotic (monaurally presented) speech, consistent with the findings in listeners with hearing loss (Reiss et al., 2016).

In comparison to previous work concerning phonological fusion (Cutting, 1975, 1976), our results showed some differences. We showed fewer fusion responses than observed previously [∼10% in the present study and ∼30% with natural speech in (Cutting, 1975)]. This may have to do with differences in the tasks. For example, the present experiment was closed-set and took place over many hours. Additional stimuli were included (e.g., same word and different vowel trials). Independently generated speech tokens were used in same word trials, and the interaural correlation of noise carriers used to generate vocoded stimuli was always zero. In the original phonological fusion experiments, stimuli were played continuously via tape and participants responded aloud in a restricted period of time. It was not described whether other word pairs were used. Subsequent studies used artificially generated speech, which would have maximized similarity between ears. Interestingly, the present results showed similar proportion of one-word responses to previous studies [58 ± 26% compared to ∼70% in (Cutting, 1975)]. Results in sections “3.2 Different vowel trials” and “3.3 Phonological fusion trials” suggest that this may be explained by shifts in attention toward one ear, leading to a one-word response. Additional data containing rhyming words are provided in Supplementary Figure 4 and suggest that the proportion of one-word responses does not change substantially across any of the vocoder conditions.

Fusion experiments generally ask listeners to report the number of sounds perceived. One alternative approach is to assess a listener’s ability to discriminate between sounds suspected to be segregated. In this case, segregation is not necessary to complete the task. This type of procedure may be more sensitive to the continuum between the perception of one versus two clear and coherent objects, where the midpoint between these possibilities is one distorted or perceptually diffuse object (e.g., Suneel et al., 2017). Using a one-interval, two-alternative forced-choice task where listeners were asked to identify whether envelope fluctuation rates were equal, Anderson et al. (2019b) showed that YNH listeners’ sensitivity to rate differences decreased when the dynamic range in one ear was reduced. The authors showed listener-dependent differences in bias of responses toward “same” or “different.” This result implies a task-relevant bias of listeners. That is, if listeners are asked to respond with one or two sounds, they might be more biased toward responding one way based upon the task and stimulus statistics. Then the high degrees of fusion in experiments might simply be indicative of poorer perceptual boundaries between features of the target and masking stimulus rather than a likelihood of perceiving one auditory object. Results from the present experiment and Anderson et al. (2019b) in YNH listeners as well as others (Ihlefeld et al., 2015; Todd et al., 2017; Anderson et al., 2022) in listeners with BiCIs suggest that temporal degradation in one ear is sufficient to interfere with cues used to segregate sound sources. In particular, the present study showed that the proportion of one-word responses decreased when the same word was presented and the proportion of two-word responses decreased when words with different vowels were presented. While the latter was a stronger effect, the present study suggests that the boundary between one and two sounds becomes less clear when stimuli are temporally degraded.

The present study investigated the effects of reduced dynamic range on speech identification. Reduced dynamic range is associated with poorer speech understanding in listeners with BiCIs (Firszt et al., 2002; Spahr et al., 2007) and simulations in NH (Loizou et al., 2000). It is also associated with poorer sensitivity to spatial cues in listeners with BiCIs (Ihlefeld et al., 2014; Todd et al., 2017) and listeners with NH (Bernstein and Trahiotis, 2011; Anderson et al., 2019b). Reducing dynamic range is similar in spirit to the spectro-temporal smearing that is thought to occur in patients. Previous experiments addressing bilateral speech understanding and showing contralateral interference in simulations of CI processing have manipulated spectro-temporal fidelity by reducing the number of frequency bands (Gallun et al., 2007; Goupell et al., 2021). Reducing the number of channels simulates spread of current that can occur with a CI, albeit in a less realistic way than vocoders that explicitly simulate current spread (Oxenham and Kreft, 2014; Croghan and Smith, 2018). Increasing the number of maxima in peak-picking, N-of-M processing strategies beyond the eight typically used in clinical practice improves speech understanding (Croghan et al., 2017). Reducing the number of spectral channels below eight is highly unlikely to be used in practice, and it is unlikely that listeners with BiCIs would be presented with such different numbers of frequency channels. It is possible that the number of “effective” channels is different between ears. Recognizing that all vocoder experiments are highly artificial, reduced or asymmetric dynamic range may be an additional problem for patients.

Aging was also associated with poorer speech identification accuracy in the present study, consistent with previous studies concerning speech recognition of older listeners with NH and CIs in background noise (Moberly et al., 2017). Similarly, open-set speech understanding accuracy in noise, but not in quiet, decreases significantly with increasing age for listeners with BiCIs (Shader et al., 2020a). Accuracy in the present study was only worse for ONH listeners when two words were presented, suggesting that effects were driven primarily by increased cognitive demand. Aging and cognition have not been associated with poorer performance in temporally-based speech perception tasks (Roque et al., 2019). Interestingly, another study showed that aging effects were greatest when open-set speech in quiet was spectrally degraded, with no differences in temporal degradation (via lowpass filtering of the envelope) for YNH compared to ONH listeners with CI simulations (Shader et al., 2020b). Older NH listeners also show poorer sensitivity to temporal speech contrasts that are level- and spectral-degradation-dependent (Goupell et al., 2017; Xie et al., 2019), suggesting that some temporal aspects of speech representation vary depending upon age. Our results suggest that task difficulty may play a key role in the emergence of aging effects on temporally based degradations of speech understanding. It was been proposed that poorer speech understanding with increasing age in listeners with CIs may be the result of reduced access to temporal envelope cues (Anderson et al., 2012). Because there was only an age effect in the best dynamic range conditions, the present results suggest that either very temporally degraded envelopes impair perception for younger and older listeners, or that aging effects are primarily driven by well-represented envelopes. Said another way, aging effects on accuracy appeared when the task was sufficiently difficult and listeners had access to the temporal envelope. Because the present study used acute exposure to simulated CI processing, differences between age groups in the 100% dynamic range condition might reflect a similar mechanism involved with poorer speech understanding of listeners with CIs who receive their implant in older age (Blamey et al., 2012).

### 4.2. Ear advantage

One interesting finding in the present study was that ear advantage, or the ear to which listeners attended, was modulated by asymmetries in dynamic range. This is consistent with the results of Goupell et al. (2021). Under symmetric conditions, listeners with NH tend to show modest effects of right-ear advantage, evidenced by greater accuracy or higher probability of reporting speech presented to the right ear compared to the left ear. This effect tends to become exaggerated as listeners get older (Westerhausen et al., 2015), though the present study only replicated this result in a subset of conditions. Thus, peripheral (e.g., temporal degradation) and central (e.g., attention-based changes with age) mechanisms may play a role in ear advantage.

Two classical hypotheses have been proposed associated with right-ear advantage, based on structural biases in the left hemisphere (Kimura, 1967) or biased attention (Kinsbourne, 1970). For listeners with NH, the attentional hypothesis seems to provide a better explanation of patterns of performance (Hiscock and Kinsbourne, 2011). It may be that shifts in attention also help to explain increased right-ear advantage associated with aging. However, it may be that structures conveying information to either side of the brain are indeed compromised for listeners with detrimental changes to the peripheral and central processing of auditory information like those who have hearing loss. Of particular concern are long periods of auditory deprivation. This conclusion is supported by one study showing that long periods of auditory deprivation in one ear are associated with speech understanding asymmetries and contralateral interference (Goupell et al., 2018). In particular, these listeners demonstrate a pattern of auditory “extinction,” where information in the poorer ear is either not perceived or ignored during simultaneous stimulation with the better ear (Deouell and Soroker, 2000). The present study and the study by Goupell et al. (2021) simulated asymmetries in the spectro-temporal fidelity of sounds in listeners with NH, which resulted in a shift in attention toward the better ear. Thus, the conclusions of a structurally based ear advantage framework may be more appropriate for understanding interaural asymmetries in speech understanding for patients with BiCIs.

**FIGURE 7:**
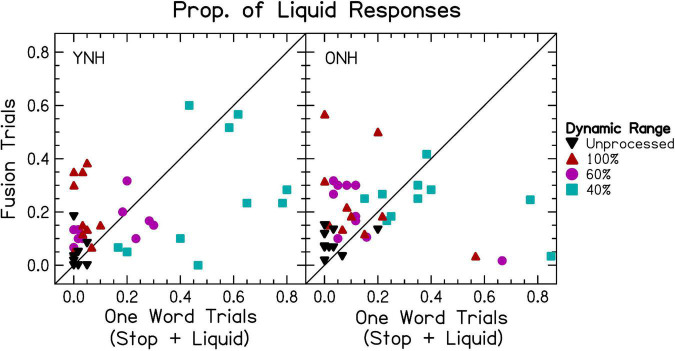
Proportion of responses containing only liquid consonants for fusion and single word trials by vocoder condition. The x- and y-axes correspond to the proportion of trials containing only liquid consonants for fusion and single word trials, respectively. The color and shape represent the vocoder condition given in the figure legend. Panels on the **(left)** and **(right)** correspond to YNH and ONH listeners, respectively.

The results from single word trials (Supplementary Figure 2) suggest that ear advantage and interference effects observed in Figure 6 could be due to consistent substitution errors (e.g., reporting a liquid when a stop and liquid were presented). In phonological fusion trials, this would be scored as a bias toward the left/right ear if the liquid matched the liquid presented, or an interference response if the liquid did not match that presented. Substituting a liquid for a stop-liquid cluster seems especially likely because of the manipulation used in the experiment. That is, decreasing the dynamic range would have smoothed the abrupt onset associated with a stop consonant. To address whether there was a consistent pattern of performance in both sets of trials, Figure 7 shows the relationship between proportion of liquid responses when stops and liquids were presented in symmetric dynamic ranges in the same word and phonological fusion trials. The results show that smaller dynamic ranges resulted in a greater number of liquid-only responses. Together with Figure 6, this suggests that listeners made a similar substitution error, reporting a liquid when both a stop and liquid were presented, especially at 100 and 60% dynamic range for both trial types. Responding with a liquid for a stimulus containing a stop and liquid in same word trials was most common at 40% dynamic range. While chance error was 0.83%, if listeners were able to understand the vowel and responded with one word, there was a 40% chance of guessing a liquid-only response. Figure 7 shows that several listeners responded with only liquids greater than 40% of the time at small dynamic ranges, suggesting that they demonstrated a consistent bias.

The vocoder manipulation used by Loizou et al. (2000) was similar to that described here, except that they reduced the overall amplitude of the envelope, which would have also decreased audibility. Our approach reduced the amplitude of the envelope and compensated by increasing the minimum, also on dB scale. This would have resulted in similar audibility, but the introduction of more prominent onsets and offsets as dynamic range became smaller. Loizou et al. (2000) had extensive data to examine how their vocoder affected speech from many different speakers, and psychophysical data to evaluate whether their predictions were correct. In our study, we had only two tokens of 15 words spoken by the same individual and accordingly wanted to avoid making generalizations. Supplementary Figure 3 indicates that listeners made very few vowel errors, suggesting that the cues needed to discriminate between the vowels here remained intact. In contrast, as can be seen in Supplementary Figure 2 and Figure 7, listeners in our study tended to confuse stop-liquid clusters with liquid consonants. The opposite pattern can be seen in the study by Loizou et al. (2000), where listeners tended to make more vowel and fewer consonant errors. This likely reflects the presence of more prominent onsets and offsets in our study.

When symmetric dynamic range was changed from 100 to 60 or 40%, it resulted in an approximately 35 or 45% decrease in the proportion of “ideal” responses, respectively (Figure 6). Approximately 10% of this decrease can be explained by an increase in “fusion” responses, corresponding to a combination of the stop and liquid words. Another 10–15% can be explained by an increase in “biased left” responses. Another 10% can be explained by an increase in “biased right” responses. The remaining decrease in “ideal” responses is explained by an increase in “interference” responses, approximately twofold in 60% and eightfold in 40%. Combined with Figure 7, these results suggest that during the fusion trials, listeners tended to report only the liquid in the left or right ear slightly more often than only the stop. Most responses from listeners still contained a stop consonant. Thus, while reporting only the liquid was the most common error, this does not mean that listeners were unable to detect the presence of the stop consonant.

Aging also had an effect on ear advantage. Older NH listeners were more likely to report the word in their poorer ear at the expense of task accuracy (Figures 3, 4). This could reflect poorer working memory or selective attention in older listeners (Roque et al., 2019). That is, compared to YNH listeners, ONH listeners were less likely to attend to the better ear but similarly likely to respond with one word when two words were presented. Stronger ear advantage effects have previously been observed (Westerhausen et al., 2015), but they were only replicated in the unprocessed conditions of the present study. Additionally, ONH listeners demonstrated more worse ear responses relative to YNH listeners when stimuli were asymmetric. It is worth noting the *p*-value (0.050) of the interaction between age and ear in symmetric dynamic ranges with different vowels and that the present study had a small sample size. Older NH listeners also showed more frequent interference responses (Figure 6), suggesting that the task was more difficult overall. Auditory spatial attention has been under investigated in ONH listeners and is a confound in BiCI studies concerning contralateral interference, e.g., (Goupell et al., 2016, 2018). One thing that can be concluded from the present study is that ONH listeners did not demonstrate the auditory extinction (i.e., lack of perceiving the worse ear when the better ear was stimulated) observed in listeners with BiCIs, suggesting that it is either acquired over asymmetric experience or a result of deafness.

### 4.3. Limitations

The greatest limitation to the generalizability of these results is probably the speech stimuli used, which were highly artificial. Speech tokens were presented with the same onset time, spoken by the same individual. It is important to note that in the original phonological fusion experiments, onset time (varied between 0 and 800 ms) played a large role in the perception of listeners. When the stop consonant preceded the liquid, listeners were even more likely to report a fused response (Cutting, 1975). In contrast, when the liquid preceded the stop, listeners were less likely to report a one-word response. When dichotic vowels are offset by as little as 1 ms, and up to 20 ms, listeners with NH and hearing loss are less likely to report a single vowel compared to when they are presented simultaneously (Eddolls et al., 2022). Unfortunately, the vowel reported in the study by Eddolls and colleagues was not included in their results, so it is not possible to determine how onset time affected the vowel being reported. Notably, there was still considerable fusion for vowels with different onset times for both groups of listeners, especially compared to differences in fundamental frequency between ears. The effect of onset time was largest and most consistent when stimuli had the same fundamental frequency, similar to the present study where all stimuli were produced by the same speaker. Stimuli in the present experiment were presented independently to each ear, rather than mixed, as would occur in the free-field. Other experiments exploring the effects of right-ear advantage have varied relative level of the sound in each ear, showing changes in the percentage of correctly reported words (Hugdahl et al., 2008; Westerhausen et al., 2009). Thus, varying the onset time and interaural level difference may help titrate ear advantage. Because there were few words in the present study, so it is possible that some of these effects were driven by specific features. It may be that other phonemes are less or more likely to result in fusion and interference than those used here. It is highly likely that listeners used any features available to distinguish between words (e.g., /d/ at the end of the set with the /ε/ vowel). Finally, listeners with CIs take a long period of experience (∼1 year) with CI stimulation before performance saturates (Blamey et al., 2012). Unilateral auditory deprivation changes the representation of auditory cues from preferring the deprived side to preferring the CI-stimulated side from brainstem to cortex (Gordon et al., 2013, 2015; Polonenko et al., 2015, 2018). Like other vocoding studies with acute exposure to CI processed speech, this approach ignores the effects of experience. In particular, because dynamic range conditions were completely randomized, it is likely that listeners would have adjusted their strategy if repeatedly exposed to the same vocoder condition.

Accordingly, the results of the present study should be treated as a proof of concept. With stimuli ideal to cause integration of auditory information, listeners were more likely to report one word that did not correspond to the word presented to either ear. One strength of the phonological fusion procedure is that it provides a spectrum of performance to assess whether the information in either ear is used, integrated, ignored, or interfered with the other ear. Future experiments may be able to use more realistic stimuli to achieve similar goals.

The present experiment successfully collected data in a remote-testing context from older and younger listeners. However, there were several challenges that occurred during testing. It was not possible to assess hearing using traditional audiometry. Instead, experimental audiometric equipment could only reliably produce sound levels of 20 dB HL, meaning that large (10–15 dB) asymmetries could not be detected if they were below 20 dB HL. Audiometric data showed asymmetric hearing responses for at least one ONH listener at high frequencies. Additionally, as monaural speech intelligibility was not measured, it was not possible to determine whether there were asymmetries in speech understanding. Further, because the experiment took so long, it is likely that listeners became tired or bored, which fit the anecdotal reports provided to the experimenter. Another limitation was that boredom or fatigue during the experiment could be misconstrued as attentional difficulties. We attempted to address this by completely randomizing experimental conditions, preventing motivation-driven decreases in performance being confounded with experimental conditions. Additionally, there were technical issues that had to be resolved during testing that would have been easier to address in the laboratory. One individual fell asleep during testing. This could be addressed in future by requiring that listeners remain in contact during the experiment, but in order to prevent burdening older listeners by requiring that they use their own equipment, it may be necessary to provide access to the internet or a phone with laboratory equipment. Remote testing has unique challenges, but it may be an equitable path forward for working with populations who are unable to travel to the laboratory, and was effective for continuing to gather data during the COVID-19 pandemic.

## 5. Summary and conclusion

Several conclusions can be drawn from the present experiment:

1. Listeners demonstrated poorer speech understanding when the dynamic range was reduced, either symmetrically or asymmetrically (Figures 3, 5), consistent with previous experiments in listeners with BiCIs (Firszt et al., 2002; Spahr et al., 2007) and simulations in NH (Loizou et al., 2000).
2. Decreased accuracy in reporting at least one word correctly was related to increased probability of one-word responses when speech with different vowels was presented to each ear (Figure 4). Speech identification accuracy was predicted by the *mean* dynamic range across ears, in contrast to previous literature suggesting that bilateral perception is dominated by the worse ear (Ihlefeld et al., 2015; Anderson et al., 2019b,^2022^) or degree of asymmetry (Yoon et al., 2011). Both findings suggest that the ears are not independent channels and interact to produce perception of speech.
3. In interaurally symmetric conditions when speech with different vowels was presented to each ear, listeners were more likely to report the stimulus in the right ear, especially when stimuli were temporally degraded (Figure 5).
4. In interaurally asymmetric conditions when speech with different vowels were presented to each ear, listeners were more likely to report the stimulus in the better ear. Older NH listeners also showed increased responses from the poorer ear with decreasing dynamic range (Figure 5).
5. When phonologically fusible pairs of words were presented to each ear, listeners were more likely to experience interference if both ears were temporally degraded (Figure 6). They were more likely to report both words correctly if both ears had a large dynamic range, and likely to report both words incorrectly if both ears had a small dynamic range (especially for older listeners).

## Data availability statement

The original contributions presented in this study are included in the article/Supplementary material, further inquiries can be directed to the corresponding author.

## Ethics statement

The studies involving human participants were reviewed and approved by University of Wisconsin-Madison Health Sciences Institution Review Board, Listeners. The patients/participants provided their digital informed consent to participate in this study.

## Author contributions

SA created the stimuli, programmed and tested all equipment, completed data collection, performed the statistical analysis, and wrote the original manuscript. SA and RL acquired funding for the study. All authors contributed to the conception and design of the study, as well as the interpretation of the results and contributed to the manuscript revision, read, and approved the submitted version.

## Footnotes

1 This code is available for free download at http://mattwinn.com/praat.html#vocoder.

2 Mixed-effects models are robust to some missing data if the data are missing at random, which can be a difficult criterion to meet. Because all trials were completely randomized within- and across-blocks, we can confirm that data were truly missing at random for ONH10 who had to terminate the experiment early.

## References

[B1] Anderson S. R. (2022). *Mechanisms that underlie poorer binaural outcomes in patients with asymmetrical hearing and bilateral cochlear implants*, Dissertation. Madison, WI: University of Wisconsin-Madison.

[B2] Anderson S. R. Kan A. Litovsky R. Y. (2019b). ‘Asymmetric temporal envelope encoding: Implications for within- and across-ear envelope comparison’. *J. Acoust. Soc. Am.* 146 1189–1206. 10.1121/1.5121423 31472559 PMC7051005

[B3] Anderson S. R. Easter K. Goupell M. J. (2019a). ‘Effects of rate and age in processing interaural time and level differences in normal-hearing and bilateral cochlear-implant listeners’. *J. Acoust. Soc. Am.* 146 3232–3254. 10.1121/1.5130384 31795662 PMC6948219

[B4] Anderson S. R. Kan A. Litovsky R. Y. (2022). Asymmetric temporal envelope encoding: Within- and across-ear envelope comparisons in listeners with bilateral cochlear implants. *J. Acoust. Soc. Am.* 152, 3294–3312. 36586876 10.1121/10.0016365 PMC9731674

[B5] Anderson S. Parbery-Clark A. White-Schwoch T. Kraus N. (2012). ‘Aging affects neural precision of speech encoding’. *J. Neurosci.* 32 14156–14164. 10.1523/JNEUROSCI.2176-12.2012 23055485 PMC3488287

[B6] Aronoff J. M. Shayman C. Prasad A. Suneel D. Stelmach J. (2015). ‘Unilateral spectral and temporal compression reduces binaural fusion for normal hearing listeners with cochlear implant simulations’. *Hear. Res.* 320 24–29. 10.1016/j.heares.2014.12.005 25549574 PMC4440320

[B7] Bakal T. A. Milvae K. D. Chen C. Goupell M. J. (2021). ‘Head shadow, summation, and squelch in bilateral cochlear-implant users with linked automatic gain controls’. *Trend. Hear.* 25 1–17. 10.1177/23312165211018147 34057387 PMC8182628

[B8] Bates D. Machler M. Bolker B. Walker S. (2015). ‘Fitting linear mixed-effects models using {lme4}’. *J. Stat. Soft.* 67 1–48. 10.18637/jss.v067.i01

[B9] Baumgärtel R. M. Hu H. Kollmeier B. Dietz M. (2017). ‘Extent of lateralization at large interaural time differences in simulated electric hearing and bilateral cochlear implant users’. *J. Acoust. Soc. Am.* 141 2338–2352. 10.1121/1.4979114 28464641

[B10] Bernstein J. G. Goupell M. J. Schuchman G. I. Rivera A. L. Brungart D. S. (2016). ‘Having two ears facilitates the perceptual separation of concurrent talkers for bilateral and single-sided deaf cochlear implantees.’. *Ear Hear.* 37 289–302. 10.1097/AUD.0000000000000284 26886027 PMC4869863

[B11] Bernstein J. G. Stakhovskaya O. A. Jensen K. K. Goupell M. J. (2020). ‘Acoustic hearing can interfere with single-sided deafness cochlear-implant speech perception’. *Ear Hear.* 41 747–761. 10.1097/AUD.0000000000000805 31584504 PMC7117997

[B12] Bernstein L. R. Trahiotis C. (2011). ‘Lateralization produced by envelope-based interaural temporal disparities of high-frequency, raised-sine stimuli: Empirical data and modeling’. *J. Acoust. Soc. Am.* 129 1501–1508. 10.1121/1.3552875 21428514 PMC3078029

[B13] Blamey P. Artieres F. Başkent D. Bergeron F. Beynon A. Burke E. (2012). ‘Factors affecting auditory performance of postlinguistically deaf adults using cochlear implants: An update with 2251 patients’. *Audiol. Neurootol.* 18 36–47. 10.1159/000343189 23095305

[B14] Bregman A. S. (1994). *Auditory scene analysis: The perceptual organization of sound.* Caimbridge, MA: Bradford Books, MIT Press, 10.1121/1.408434

[B15] Brungart D. S. Simpson B. D. (2002). ‘Within-ear and across-ear interference in a cocktail-party listening task’. *J. Acoust. Soc. Am.* 112 2985–2995. 10.1121/1.1512703 12509020

[B16] Cherry E. C. (1953). ‘Some experiments on the recognition of speech, with one and with two ears’. *J. Acoust. Soc. Am.* 25 975–979. 10.1121/1.1907229

[B17] Croghan N. B. H. Smith Z. M. (2018). ‘Speech understanding with various maskers in cochlear-implant and simulated cochlear-implant hearing: Effects of spectral resolution and implications for masking release’. *Trend. Hear.* 22 1–13. 10.1177/2331216518787276 30022730 PMC6053854

[B18] Croghan N. B. H. Duran S. I. Smith Z. M. (2017). ‘Re-examining the relationship between number of cochlear implant channels and maximal speech intelligibility’. *J. Acoust. Soc. Am.* 142 EL537–EL543. 10.1121/1.5016044 29289062

[B19] Cutting J. E. (1975). ‘Aspects of phonological fusion’. *J. Exp. Psychol. Hum. Percept. Perform.* 104 105–120. 10.1037/0096-1523.1.2.105 1194864

[B20] Cutting J. E. (1976). ‘Auditory and linguistic processes in speech perception: Inferences from six fusions in dichotic listening’. *Psych. Rev.* 83 114–140. 10.1037/0033-295X.83.2.114 769016

[B21] Darwin C. J. (1981). ‘Perceptual grouping of speech components differing in fundamental frequency and onset-time’. *Q. J. Exp. Psychol.* 33 185–207. 10.1080/14640748108400785

[B22] Deouell L. Y. Soroker N. (2000). ‘What is extinguished in auditory extinction?’. *Neuroreport* 11 3059–3062. 10.1097/00001756-200009110-00046 11006994

[B23] Drullman R. Festen J. M. Plomp R. (1994). ‘Effect of temporal envelope smearing on speech reception’. *J. Acoust. Soc. Am.* 95 1053–1064. 10.1121/1.408467 8132899

[B24] Durlach N. I. (1963). ‘Equalization and cancellation theory of binaural masking-level differences’. *J. Acoust. Soc. Am.* 35 1206–1218. 10.1121/1.1918675

[B25] Eddolls M. S. Molis M. R. Reiss L. A. J. (2022). ‘Onset asynchrony: Cue to aid dichotic vowel segregation in listeners with normal hearing and hearing loss’. *J. Speech Lang. Hear. Res.* 65 2709–2719. 10.1044/2022_jslhr-21-00411 35728021 PMC9584133

[B26] Firszt J. B. Chambers R. D. Kraus N. (2002). ‘Neurophysiology of cochlear implant users II: Comparison among speech perception, dynamic range, and physiological measures’. *Ear Hear.* 23 516–531. 10.1097/00003446-200212000-00003 12476089

[B27] Frank T. (1997). ‘ANSI update: Specification of audiometers’. *Am. J. Audiol.* 6 29–32.

[B28] Gallun F. J. Mason C. R. Kidd G. J. (2007). ‘The ability to listen with independent ears’. *J. Acoust. Soc. Am.* 122 2814–2825. 10.1121/1.2780143 18189571

[B29] Gallun F. J. McMillan G. P. Molis M. R. Kampel S. D. Dann S. M. Konrad-Martin D. L. (2014). ‘Relating age and hearing loss to monaural, bilateral, and binaural temporal sensitivity’. *Front. Neuro.* 8:1–14. 10.3389/fnins.2014.00172 25009458 PMC4070059

[B30] Gordon K. A. Jiwani S. Papsin B. C. (2013). ‘Benefits and detriments of unilateral cochlear implant use on bilateral auditory development in children who are deaf’. *Front Psychol* 4:719. 10.3389/fpsyg.2013.00719 24137143 PMC3797443

[B31] Gordon K. Henkin Y. Kral A. (2015). ‘Asymmetric hearing during development: The aural preference syndrome and treatment options’. *Pediatrics* 136 141–153. 10.1542/peds.2014-3520 26055845

[B32] Goupell M. J. Eisenberg D. DeRoy Milvae K. (2021). ‘Dichotic listening performance with cochlear-implant simulations of ear asymmetry is consistent with difficulty ignoring clearer speech’. *Atten. Percept. Psychophys.* 83 2083–2101. 10.3758/s13414-021-02244-x 33782914 PMC8480144

[B33] Goupell M. J. Gaskins C. R. Shader M. J. Walter E. P. Anderson S. Gordon-Salant S. (2017). ‘Age-related differences in the processing of temporal envelope and spectral cues in a speech segment’. *Ear Hear.* 38 e335–e342. 10.1097/AUD.0000000000000447 28562426 PMC5659932

[B34] Goupell M. J. Kan A. Litovsky R. Y. (2016). ‘Spatial attention in bilateral cochlear-implant users’. *J. Acoust. Soc. Am.* 140 1652–1662. 10.1121/1.4962378 27914414 PMC5848865

[B35] Goupell M. J. Noble J. H. Phatak S. A. Kolberg E. Cleary M. Stakhovskaya O. A. (2022). ‘Computed-tomography estimates of interaural mismatch in insertion depth and scalar location in bilateral cochlear-implant users’. *Otol. Neurotol.* 43 666–675. 10.1097/MAO.0000000000003538 35761459 PMC9245128

[B36] Goupell M. J. Stakhovskaya O. A. Bernstein J. G. W. (2018). ‘Contralateral interference caused by binaurally presented competing speech in adult bilateral cochlear-implant users’. *Ear Hear.* 39 110–123. 10.1097/AUD.0000000000000470 28787316 PMC5741461

[B37] Goupell M. J. Stoelb C. Kan A. Litovsky R. Y. (2013). ‘Effect of mismatched place-of-stimulation on the salience of binaural cues in conditions that simulate bilateral cochlear-implant listening’. *J. Acoust. Soc. Am.* 133 2272–2287. 10.1121/1.4792936 23556595 PMC3631247

[B38] Greenwood D. D. (1990). ‘A cochlear frequency-position function for several species—29 years later’. *J. Acoust. Soc. Am.* 87 2592–2605. 10.1121/1.399052 2373794

[B39] Hiscock M. Kinsbourne M. (2011). ‘Attention and the right-ear advantage: What is the connection?’. *Brain Cogn.* 76 263–275. 10.1016/j.bandc.2011.03.016 21507543

[B40] Hugdahl K. Westerhausen R. Alho K. Medvedev S. Hämäläinen H. (2008). ‘The effect of stimulus intensity on the right ear advantage in dichotic listening’. *Neurosci. Lett.* 431 90–94. 10.1016/j.neulet.2007.11.046 18162310

[B41] Ihlefeld A. Carlyon R. P. Kan A. Churchill T. H. Litovsky R. Y. (2015). ‘Limitations on monaural and binaural temporal processing in bilateral cochlear implant listeners’. *J. Assoc. Res. Otolaryngol.* 16 641–652. 10.1007/s10162-015-0527-7 26105749 PMC4569611

[B42] Ihlefeld A. Kan A. Litovsky R. Y. (2014). ‘Across-frequency combination of interaural time difference in bilateral cochlear implant listeners’. *Front. Syst. Neurosci.* 8:22. 10.3389/fnsys.2014.00022 24653681 PMC3949319

[B43] Kan A. Goupell M. J. Litovsky R. Y. (2019). ‘Effect of channel separation and interaural mismatch on fusion and lateralization in normal-hearing and cochlear-implant listeners’. *J. Acoust. Soc. Am.* 146 1448–1463. 10.1121/1.5123464 31472555 PMC6713556

[B44] Kan A. Stoelb C. Litovsky R. Y. Goupell M. J. (2013). ‘Effect of mismatched place-of-stimulation on binaural fusion and lateralization in bilateral cochlear-implant users’. *J. Acoust. Soc. Am.* 134 2923–2936. 10.1121/1.4820889 24116428 PMC3799729

[B45] Kenward M. G. Roger J. H. (1997). ‘Small sample inference for fixed effects from restricted maximum likelihood’. *Biometrics* 53 983–997. 10.2307/2533558 9333350

[B46] Kimura D. (1967). ‘Functional asymmetry of the brain in dichotic listening’. *Cortex* 3 163–178. 10.1016/s0010-9452(67)80010-8

[B47] Kinsbourne M. (1970). ‘The cerebral basis of lateral asymmetries in attention’. *Acta Psychol. (Amst)* 33 193–201. 10.1016/0001-6918(70)90132-0 5445964

[B48] Kuznetsova A. Brockhoff P. B. Christensen R. H. B. (2017). ‘lmerTest package: Tests in linear mixed effects models’. *J. Stat. Softw.* 82 1–26. 10.18637/jss.v082.i13

[B49] Lenth R. V. (2022). *emmeans: Estimated marginal means, aka least-squares means’. CRAN.* Available online at: https://cran.r-project.org/package=emmeans (accessed December 24, 2022).

[B50] Litovsky R. Y. Colburn H. S. Yost W. A. Guzman S. J. (1999). ‘The precedence effect’. *J. Acoust. Soc. Am.* 106 1633–1654. 10.1016/0378-5955(83)90002-3 10530009

[B51] Litovsky R. Y. Jones G. L. Agrawal S. Hoesel R v (2010). ‘Effect of age at onset of deafness on binaural sensitivity in electric hearing in humans’. *J. Acoust. Soc. Am.* 127 400–414. 10.1121/1.3257546 20058986 PMC2821168

[B52] Litovsky R. Parkinson A. Arcaroli J. Sammeth C. (2006). ‘Simultaneous bilateral cochlear implantation in adults: A multicenter clinical study’. *Ear Hear.* 27 714–731. 10.1097/01.aud.0000246816.50820.42 17086081 PMC2651401

[B53] Loizou P. C. Dorman M. F. Fitzke J. (2000). ‘The effect of reduced dynamic range on speech understanding: Implications for patients with cochlear implants’. *Ear Hear.* 21 25–31. 10.1097/00003446-200002000-00006 10708071

[B54] Loizou P. C. Hu Y. Litovsky R. Yu G. Peters R. Lake J. (2009). ‘Speech recognition by bilateral cochlear implant users in a cocktail-party setting’. *J. Acoust. Soc. Am.* 125 372–383. 10.1121/1.3036175 19173424 PMC2676860

[B55] Long C. J. Eddington D. K. Colburn H. S. Rabinowitz W. M. (2003). ‘Binaural sensitivity as a function of interaural electrode position with a bilateral cochlear implant user’. *J. Acoust. Soc. Am.* 114 1565–1574. 10.1121/1.1603765 14514210

[B56] Long C. J. Holden T. A. McClelland G. H. Parkinson W. S. Shelton C. Kelsall D. C. (2014). ‘Examining the electro-neural interface of cochlear implant users using psychophysics. CT scans, and speech understanding’, *J. Assoc. Res. Otolaryngol.* 15 293–304. 10.1007/s10162-013-0437-5 24477546 PMC3946134

[B57] Moberly A. C. Harris M. S. Boyce L. Nittrouer S. (2017). ‘Speech recognition in adults with cochlear implants: The effects of working memory, phonological sensitivity, and aging’. *J. Speech Lang. Hear. Res.* 60 1046–1061. 10.1044/2016_JSLHR-H-16-0119 28384805 PMC5548076

[B58] Mosnier I. Sterkers O. Bebear J. Godey B. Robier A. Deguine O. (2009). ‘Speech performance and sound localization in a complex noisy environment in bilaterally implanted adult patients’. *Audiol. Neurootol.* 14 106–114. 10.1159/000159121 18832816

[B59] Oxenham A. J. Kreft H. A. (2014). ‘Speech perception in tones and noise via cochlear implants reveals influence of spectral resolution on temporal processing’. *Trend. Hear.* 18 1–14. 10.1177/2331216514553783 25315376 PMC4227666

[B60] Polonenko M. J. Papsin B. C. Gordon K. A. (2015). ‘The effects of asymmetric hearing on bilateral brainstem function: Findings in children with bimodal (electric and acoustic) hearing’. *Audiol. Neurootol.* 20(Suppl. 1), 13–20. 10.1159/000380743 25998954

[B61] Polonenko M. J. Papsin B. C. Gordon K. A. (2018). ‘Limiting asymmetric hearing improves benefits of bilateral hearing in children using cochlear implants’. *Sci. Rep.* 8 1–17. 10.1038/s41598-018-31546-8 30181590 PMC6123397

[B62] Pumplin J. (1985). ‘Low-noise noise’. *J. Acoust. Soc. Am.* 78 100–104. 10.1121/1.392571

[B63] Rao R. P. N. Ballard D. H. (1999). ‘Predictive coding in the visual cortex: A functional interpretation of some extra-classical receptive-field effects’. *Nat. Neurosci.* 2 79–87. 10.1038/4580 10195184

[B64] Reeder R. M. Firszt J. B. Holden L. K. Strube M. J. (2014). ‘A longitudinal study in adults with sequential bilateral cochlear implants: Time course for individual ear and bilateral performance’. *J. Speech Lang. Hear. Res.* 57 1108–1126. 10.1044/2014 24686778 PMC4057980

[B65] Reiss L. A. J. Molis M. R. (2021). ‘Abnormal fusion of dichotic vowels across different fundamental frequencies in hearing-impaired listeners: An alternative explanation for difficulties with speech in background talkers’. *J. Assoc. Res. Otolaryngol.* 22 443–461. 10.1007/s10162-021-00790-7 33877470 PMC8329143

[B66] Reiss L. A. J. Eggleston J. L. Walker E. P. Oh Y. (2016). ‘Two ears are not always better than one: Mandatory vowel fusion across spectrally mismatched ears in hearing-impaired listeners’. *J. Assoc. Res. Otolaryngol.* 17 341–356. 10.1007/s10162-016-0570-z 27220769 PMC4940290

[B67] Reiss L. A. Fowler J. R. Hartling C. L. Oh Y. (2018). ‘Binaural pitch fusion in bilateral cochlear implant users’. *Ear Hear.* 39 390–397. 10.1097/AUD.0000000000000497 28945657 PMC5821581

[B68] Reiss L. A. Ito R. A. Eggleston J. L. Liao S. Becker J. J. Lakin C. E. (2015). ‘Pitch adaptation patterns in bimodal cochlear implant users: Over time and after experience’. *Ear Hear.* 36 e23–e34. 10.1097/AUD.0000000000000114 25319401 PMC4336615

[B69] Roque L. Karawani H. Gordon-Salant S. Anderson S. (2019). ‘Effects of age, cognition, and neural encoding on the perception of temporal speech cues’. *Front. Neuro.* 13:1–15. 10.3389/fnins.2019.00749 31379494 PMC6659127

[B70] Scharf B. (1974). “Localization of unlike tones from two loudspeakers,” in *Sensation and Measurement*, eds Moskowitz H. R. Scharf B. Stevens J. C. (Dordrecht: Springer), 309–314. 10.1007/978-94-010-2245-3_30

[B71] Shader M. J. Nguyen N. Cleary M. Hertzano R. Eisenman D. J. Anderson S. (2020a). ‘Effect of stimulation rate on speech understanding in older cochlear-implant users’. *Ear Hear.* 41 640–651. 10.1097/AUD.0000000000000793 31702596 PMC7190412

[B72] Shader M. J. Yancey C. M. Gordon-Salant S. Goupell M. J. (2020b). ‘Spectral-temporal trade-off in vocoded sentence recognition: Effects of age, hearing thresholds, and working memory’. *Ear Hear.* 41 1226–1235. 10.1097/AUD.0000000000000840 32032222 PMC7415490

[B73] Shannon R. V. Zeng F. G. Kamath V. Wygonski J. Ekelid M. (1995). ‘Speech recognition with primarily temporal cues’. *Science* 270 303–304. 10.1126/science.270.5234.303 7569981

[B74] Shepherd R. K. Hardie N. A. (2001). ‘Deafness-induced changes in the auditory pathway: Implications for cochlear implants’. *Audiol. Neurootol.* 6 305–318. 10.1159/000046843 11847461

[B75] Shinn-Cunningham B. G. (2008). ‘Object-based auditory and visual attention’. *Trends Cogn. Sci.* 12 182–186. 10.1016/j.tics.2008.02.003 18396091 PMC2699558

[B76] Shinn-Cunningham B. Best V. Lee A. K. C. (2017). “Auditory object formation and selection,” in *The auditory system at the cocktail party. Springer handbook of auditory research*, 60th Edn, eds Middlebrooks J. C. (Cham: Springer), 7–40. 10.1007/978-3-319-51662-2_2

[B77] Spahr A. J. Dorman M. F. Loiselle L. H. (2007). ‘Performance of patients using different cochlear implant systems: Effects of input dynamic range’. *Ear Hear.* 28 260–275. 10.1097/AUD.0b013e3180312607 17496675

[B78] Staisloff H. E. Lee D. H. Aronoff J. M. (2016). ‘Perceptually aligning apical frequency regions leads to more binaural fusion of speech in a cochlear implant simulation’. *Hear. Res.* 337 59–64. 10.1016/j.heares.2016.05.002 27208791 PMC5014763

[B79] Suneel D. Staisloff H. Shayman C. S. Stelmach J. Aronoff J. M. (2017). ‘Localization performance correlates with binaural fusion for interaurally mismatched vocoded speech’. *J. Acoust. Soc. Am.* 142 EL276–EL280. 10.1121/1.5001903 28964063 PMC5724736

[B80] Todd A. E. Goupell M. J. Litovsky R. Y. (2017). ‘The relationship between intensity coding and binaural sensitivity in adults with cochlear implants’. *Ear Hear.* 38 e128–e141. 10.1097/AUD.0000000000000382 27787393 PMC5322240

[B81] van den Brink G. Sintnicolaas K. van Stam W. S. (1976). ‘Dichotic pitch fusion’. *J. Acoust. Soc. Am.* 59 1471–1476. 10.1121/1.380989 939880

[B82] van Hoesel R. J. M. Clark G. M. (1995). ‘Fusion and lateralization study with two binaural cochlear implant patients’. *Ann. Otol. Rhinol. Laryngol.* 104 233–235. 7668650

[B83] van Hoesel R. J. M. Clark G. M. (1997). ‘Psychophysical studies with two binaural cochlear implant subjects’. *J. Acoust. Soc. Am.* 102 495–507. 10.1121/1.419611 9228813

[B84] Westerhausen R. Bless J. Kompus K. (2015). ‘Behavioral laterality and aging: The free-recall dichotic-listening right-ear advantage increases with age’. *Dev. Neuropsychol.* 40 313–327. 10.1080/87565641.2015.1073291 26285097

[B85] Westerhausen R. Moosmann M. Alho K. Medvedev S. Hämäläinen H. Hugdahl K. (2009). ‘Top-down and bottom-up interaction: Manipulating the dichotic listening ear advantage’. *Brain Res.* 1250 183–189. 10.1016/j.brainres.2008.10.070 19028471

[B86] Whitmer W. M. Seeber B. U. Akeroyd M. A. (2014). ‘The perception of apparent auditory source width in hearing-impaired adults’. *J. Acoust. Soc. Am.* 135 3548–3559. 10.1121/1.4875575 24907818 PMC4152617

[B87] Woods K. J. Siegel M. H. Traer J. McDermott J. H. (2017). ‘Headphone screening to facilitate web-based auditory experiments’. *Atten. Percept. Psychophys.* 79 2064–2072. 10.3758/s13414-017-1361-2 28695541 PMC5693749

[B88] Xie Z. Gaskins C. R. Shader M. J. Gordon-Salant S. Anderson S. Goupell M. J. (2019). ‘Age-related temporal processing deficits in word segments in adult cochlear-implant users’. *Trend. Hear.* 23:2331216519886688. 10.1177/2331216519886688 31808373 PMC6900735

[B89] Yoon Y. Li Y. Kang H. Fu Q. (2011). ‘The relationship between binaural benefit and difference in unilateral speech recognition performance for bilateral cochlear implant users’. *Int. J. Audiol.* 50 554–565. 10.3109/14992027.2011.580785 21696329 PMC3160169

