# Supplementary material for: Interaural asymmetry of dynamic range: Abnormal fusion, bilateral interference, and shifts in attention
Source: Front Neurosci. 2023 Jan 9;16:1018190. doi: 10.3389/fnins.2022.1018190 (PMC9869277; doi:10.3389/fnins.2022.1018190)
Supplement: Supplementary file 1 [file Image_1.pdf]

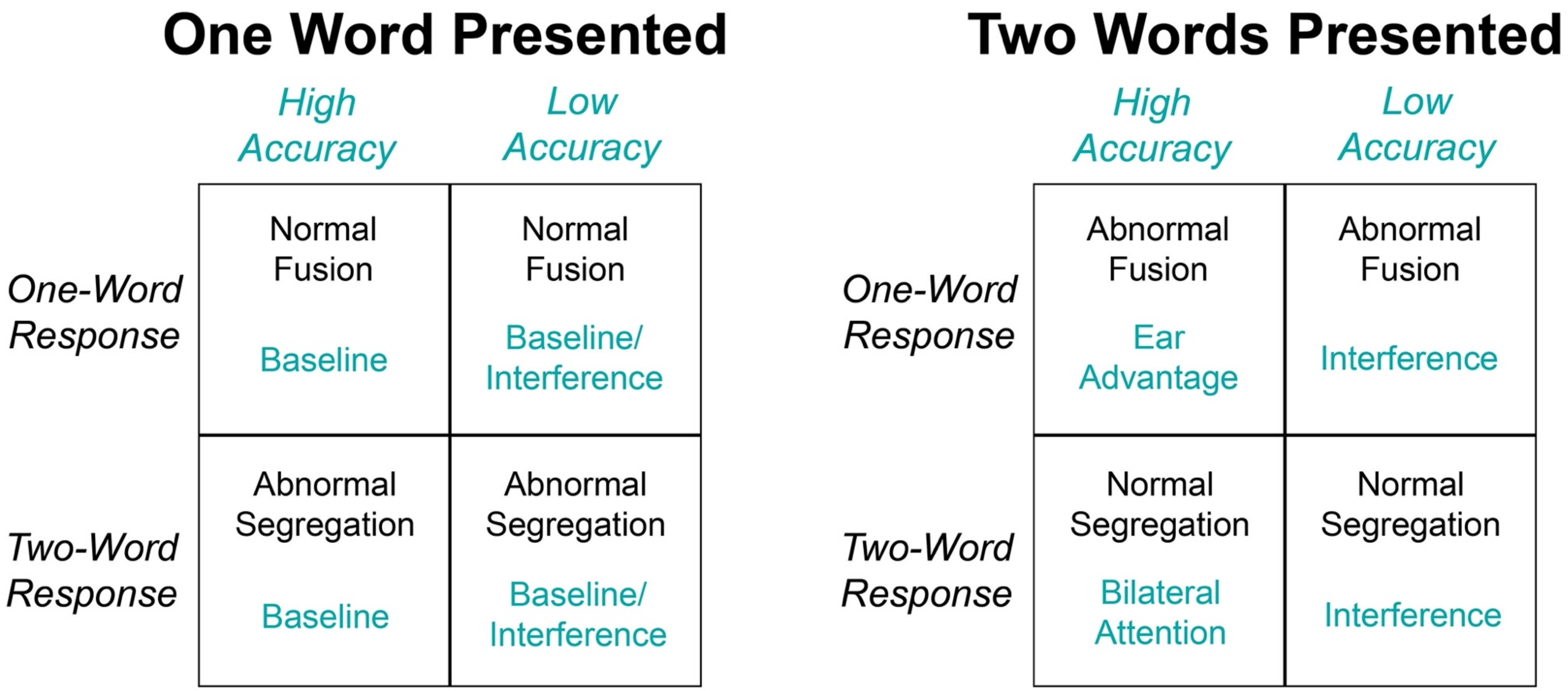

SUPPLEMENTARY FIG. 1. Graphical illustration of interpretation of results. Each panel represents a different possible outcome and its corresponding interpretation in the present experiment. Accuracy of responses is shown in turquoise and number of responded words is shown in black. When one word was presented, interference would be implied if asymmetric vocoder conditions resulted in a decrease in accuracy, which may or may not be accompanied by a change in the number of words responded. The panels on the left apply to Sec. 3.1 (Fig. 2) of the Results. Note that a two-word response was considered incorrect. See also Supplementary Fig. 2 for the patterns of confusions including one- and two-word responses. The set of panels on the right apply to Sec. 3.2 and 3.3 (Fig. 3; Supp. Fig. 4) of the Results. See also Fig. 5 for a breakdown of the different patterns of results on phonological fusion trials.

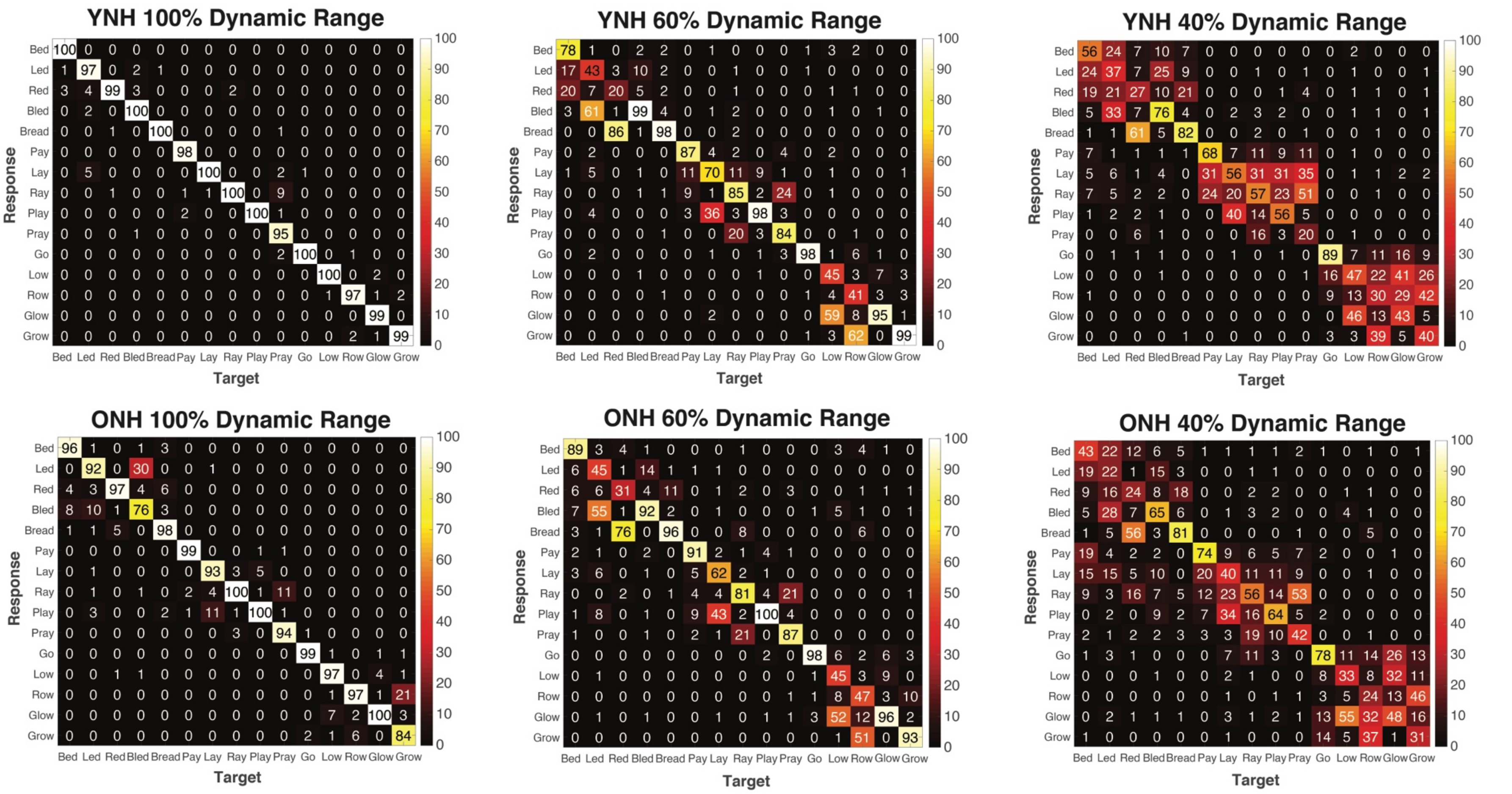

SUPPLEMENTARY FIG. 2. Single word confusion matrices. Each panel corresponds to a different vocoder condition and group given at the top. The x-axis represents the word presented. The y-axis represents the word(s) responded. Responses for all individuals are shown for all listeners. The color and number within each cell represent the number of responses for a given target.

Trials with one- and two-word responses were both included. The results suggest that, for 60% dynamic range, listeners were more likely to confuse speech beginning with a liquid with stimuli containing a stop-liquid cluster with the same liquid. The same pattern of errors can be seen for 40% dynamic range, though listeners were more likely to more likely to confuse all words sharing the same vowel, and more likely to make a vowel confusion compared to 100% and 60%. Additionally, listeners in the 40% dynamic range conditions seemed to report words

beginning with a liquid when a stop only was presented. Patterns of confusions are explored further in the discussion.

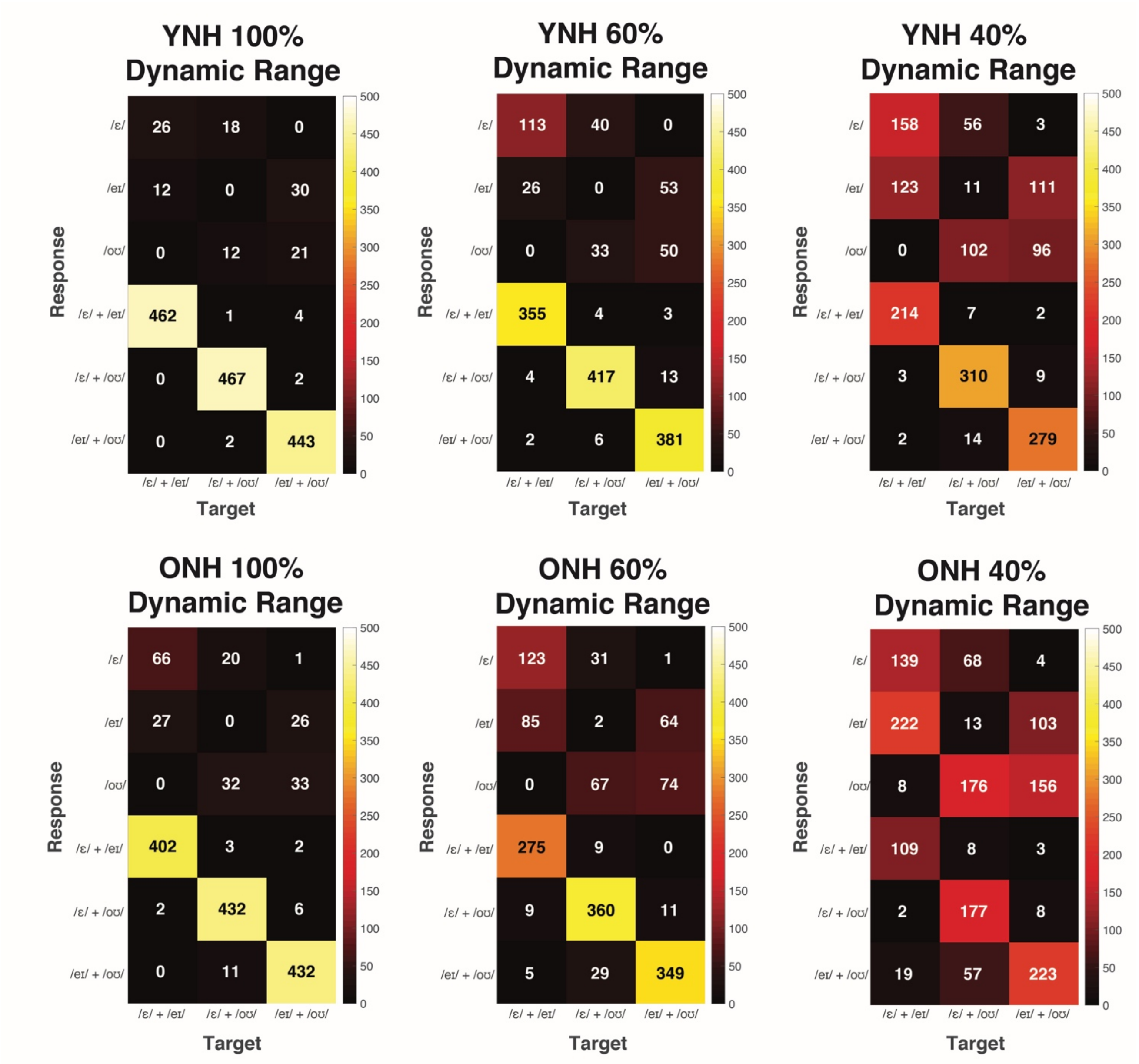

SUPPLEMENTARY FIG. 3. Different vowel confusion matrices. Each panel corresponds to a different vocoder condition and age group given at the top. The x-axis represents the vowels of the words presented. The y-axis represents the vowel(s) of the word(s) responded. Responses for all individuals are shown for all listeners. The color and number within each cell represent the number of responses for a given target.

We wanted to titrate the types of errors made in each symmetric vocoder condition. There were a total of 75, two-word response combinations as well as 15 one-word response possibilities. Thus, a confusion matrix would be difficult to show with every possible combination. Instead, capitalizing on the small number of vowel errors made with single word trials, this figure shows vowel confusion matrices for interaurally symmetric vocoder trials. There were only three possible vowel combinations on each trial, but single vowel responses were also considered. As can be seen from this figure, vowel errors were very rare. When listeners reported a single vowel, this usually corresponded to one of the vowels presented in one ear. The /ɛ/ and /eɪ/ pairs were the most likely to

result in singular vowel responses. This may have to do with the fact that the /ɛ/ set had an additional /d/ cue at the end of each word.

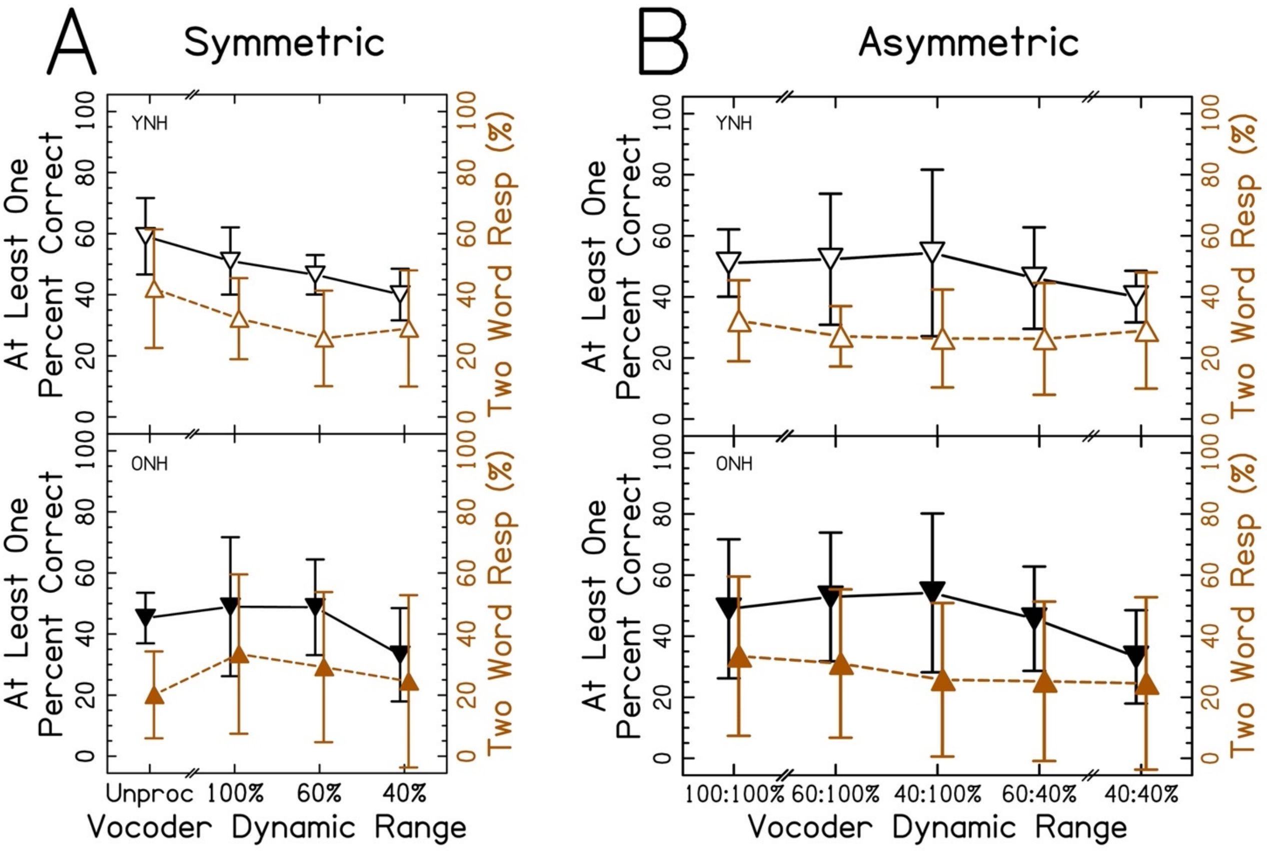

SUPPLEMENTARY FIG. 4. Number of words responded for (A) interaurally symmetric and (B) interaurally asymmetric vocoder conditions in rhyming word trials. The x-axis corresponds to the vocoder condition. The y-axis represents the percentage of trials with at least one word accurately identified (△ shown in black) and the percentage of two-word responses (▽ shown in brown). Open and closed shapes represent YNH and ONH listeners, respectively.

An ANOVA with speech identification accuracy as the dependent variable revealed significant effects of vocoder condition [$\chi^2(6) = 170.927$, $p < 0.0001$], but not age group [$\chi^2(1) = 0.655$, $p = 0.200$]. There was also a significant vocoder condition × age group [$\chi^2(6) = 40.398$, $p < 0.0001$] interaction. Similarly, an ANOVA with proportion of two-word responses as the dependent variable revealed significant effects of vocoder condition [$\chi^2(6) = 53.679$, $p < 0.0001$], but not age group [$\chi^2(1) = 0.346$, $p = 0.556$]. There was also a significant vocoder condition × age group [$\chi^2(6) = 119.850$, $p < 0.0001$] interaction.
